# In‐depth interrogation of protein thermal unfolding data with MoltenProt

**DOI:** 10.1002/pro.3986

**Published:** 2020-11-21

**Authors:** Vadim Kotov, Georg Mlynek, Oliver Vesper, Marina Pletzer, Jiri Wald, Celso M. Teixeira‐Duarte, Herve Celia, Maria Garcia‐Alai, Stephan Nussberger, Susan K. Buchanan, João H. Morais‐Cabral, Christian Loew, Kristina Djinovic‐Carugo, Thomas C. Marlovits

**Affiliations:** ^1^ Centre for Structural Systems Biology (CSSB) Hamburg Germany; ^2^ Institute for Structural and Systems Biology University Medical Center Hamburg‐Eppendorf (UKE) Hamburg Germany; ^3^ German Electron Synchrotron Centre (DESY) Hamburg Germany; ^4^ Department of Structural and Computational Biology, Max Perutz Labs Vienna University of Vienna Vienna Austria; ^5^ Instituto de Investigação e Inovação em Saúde (i3S) and Instituto de Biologia Molecular e Celular (IBMC) Universidade do Porto Porto Portugal; ^6^ Laboratory of Molecular Biology, National Institute of Diabetes & Digestive & Kidney Diseases National Institutes of Health Bethesda Maryland USA; ^7^ Department of Biophysics, Institute of Biomaterials and Biomolecular Systems University of Stuttgart Stuttgart Germany; ^8^ Department of Biochemistry, Faculty of Chemistry and Chemical Technology University of Ljubljana Ljubljana Slovenia; ^9^ European Molecular Biology Laboratory (EMBL) Hamburg Unit Hamburg Germany

**Keywords:** buffer optimization, high‐throughput screening, melting temperature, MoltenProt, NanoDSF, protein unfolding, thermostability

## Abstract

Protein stability is a key factor in successful structural and biochemical research. However, the approaches for systematic comparison of protein stability are limited by sample consumption or compatibility with sample buffer components. Here we describe how miniaturized measurement of intrinsic tryptophan fluorescence (NanoDSF assay) in combination with a simplified description of protein unfolding can be used to interrogate the stability of a protein sample. We demonstrate that improved protein stability measures, such as apparent Gibbs free energy of unfolding, rather than melting temperature T_m_, should be used to rank the results of thermostability screens. The assay is compatible with protein samples of any composition, including protein complexes and membrane proteins. Our data analysis software, MoltenProt, provides an easy and robust way to perform characterization of multiple samples. Potential applications of MoltenProt and NanoDSF include buffer and construct optimization for X‐ray crystallography and cryo‐electron microscopy, screening for small‐molecule binding partners and comparison of effects of point mutations.

## 1 INTRODUCTION

**FIGURE 1:**
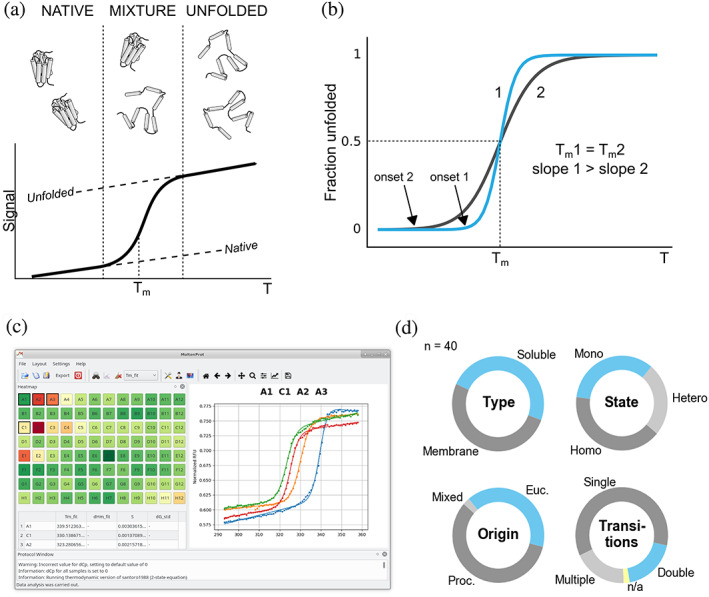
MoltenProt is a fast and efficient tool for analysis of protein unfolding data. (a) A typical thermal unfolding assay. Signal increases as a protein unfolds with increasing temperature (T). The inflection point of the curve (melting temperature T_m_) indicates the temperature where 50% of protein molecules are in an unfolded state. Dashed lines indicate linearly extrapolated baselines, that is, temperature dependence of native or unfolded state fluorescence. Vertical dashed lines denote the transition region (1%–99% of protein molecules are unfolded). (b) Interpretation of slope values for thermal unfolding curves. Curves 1 and 2 have identical T_m_, but curve 1 has a steeper (higher) slope. A steeper slope translates to a later onset of unfolding, that is, the temperature at which an arbitrary fraction of protein molecules becomes unfolded. Thus, the folded state of the protein in condition 1 is more resistant to heat, and it could be considered more stabilizing than condition 2. Importantly, these conditions are identical in terms of T_m_. (c) A screenshot of the MoltenProt GUI. Left‐hand side presents the samples in a 96‐well format color‐coded for a chosen fit parameter (e.g., ΔG_u_°′, T_m_or ΔH_m_). Right‐hand side displays one or more curves for comparison and assessment of fit quality. (d) Diversity of tested protein samples (*n* = 40) by type, oligomeric state, origin and the number of unfolding transitions. State indicates whether a protein sample forms oligomers (homooligomers or heterooligomers) or is a monomeric protein. Mixed origin indicates protein complexes with subcomponents from different species (e.g., antigen–antibody complex)

Functional, biochemical or structural analyses of proteins and protein complexes require stable and monodisperse protein samples. A widely used proxy for overall protein stability is thermostability.^1^ In a typical assay (Figure 1(a)) a protein sample is gradually heated up, and the fraction of unfolded molecules is monitored using a spectroscopic or calorimetric readout. The inflection point of the unfolding curve, also known as the melting temperature (T_m_), is determined from the experimental derivative or curve fitting. Protein samples with higher T_m_ tend to be more stable and perform well in biochemical and structural biology research.^1^, ^2^, ^3^, ^4^ Thermostability measurements are robust, consume low amounts of protein material and do not require expensive equipment. Furthermore, resulting T_m_ values are highly reproducible^5^ and agree across orthogonal assays, such as differential scanning calorimetry (DSC)^6^ or circular dichroism measurements (CD).^4^ This makes thermostability an excellent choice for screening buffer conditions and optimization of protein constructs.^4^, ^7^, ^8^, ^9^

To date, high‐throughput approaches for thermostability measurements, such as Thermofluor^10^ or NanoDSF,^11^, ^12^ were primarily focused on determination of T_m_. This approach was criticized earlier,^7^ and the importance of the slope of the unfolding curve was highlighted (Figure 1(b)). Indeed, apart from the baselines, the simplest sigmoidal curve can be described by two values: (a) inflection point (T_m_) and (b) the slope of the unfolding curve, that is, how steep or flat the transition is. Characterizing these curves with two parameters also presents a challenge on how to rank the results,^13^ since the parameters may have different weights and changes in parameters may contradict each other. To address this issue, Chari et al.^7^ proposed an empirical “hierarchical sorting” procedure, where the results are sequentially sorted and filtered by three curve parameters (quality of the fit, slope, and T_m_). This approach assigns discrete numbers to final hits, so the differences between sample stabilities are qualitative and not quantitative. Furthermore, the sorting is done in a specific sequence of steps, thus assigning arbitrary weights to parameters. Finally, “hierarchical sorting” assesses ¼ of the data, disregarding the majority of the stability‐related information and was thus recommended exclusively as a “qualitative guideline”.^7^

In this work we introduce and validate a quantitative approach for ranking results of thermal unfolding screens and describe a software package, MoltenProt, that streamlines the data analysis pipeline and makes it easy to use. In addition, we discuss several case studies to prove the utility of MoltenProt in structural and biochemical research.

### 1.1 *Overview of available techniques*

Diverse assays are available to monitor chemical and thermal unfolding of proteins and obtain thermodynamic parameters (Table S1). DSC provides direct estimates of protein ΔC_p_, ΔH_m_, and T_m_, however, the assay consumes high amounts of protein material (2.5–2,500 μg for a single measurement, depending on the sample) and cannot be efficiently parallelized. Furthermore, unstable buffer components, such as dithiothreitol (DTT) and tris(2‐carboxyethyl)phosphine (TCEP), degrade during heating and distort the signal by affecting the buffer baseline.^14^ CD measurements take the advantage of chirality in amino acids to quantify the secondary structure content in proteins.^15^ CD is routinely used to monitor thermal and chemical unfolding of proteins,^16^ however, the assay requires all buffer components to be optically inactive in the ultraviolet (UV) region.^17^ In particular, chloride ions, imidazole, DTT, dimethylsulfoxide (DMSO), glycerol, and several detergents absorb strongly in the far‐UV region and should be avoided. Furthermore, CD was not adapted for high‐throughput analysis and sample consumption can be high (2.25–2,250 μg for a single measurement, depending on the sample). High‐throughput assays to assess protein thermostability include Thermofluor^10^, ^18^ and the 7‐diethylamino‐3‐(4′‐maleimidylphenyl)‐4‐methylcoumarin (CPM) dye assay.^19^ These assays use a fluorescent dye to report protein unfolding: in Thermofluor, fluorescence increases when the dye binds to hydrophobic regions exposed during protein unfolding, while the CPM dye is only fluorescent upon reaction with exposed thiol groups. Protein sample is dispensed in a microplate, and efficient parallelization can be easily implemented. For both assays as low as 2–4 μg of protein per sample is sufficient, however, the increase in throughput is achieved by lower quality and information content of the signal. Previous work^7^ reported up to 30% relative error in ΔH_m_ estimation and 0.5 K error for T_m_ obtained with a modified Thermofluor assay. SYPRO Orange, the most commonly used fluorescent dye for Thermofluor, is not compatible with detergents and a number of other buffer components^20^ as well as with heme proteins or proteins tagged with green fluorescent protein (GFP). Other dyes, such as 1‐anilino‐naphthalene‐8‐sulfonate (ANS) and GloMelt (manufactured by Biotinum, San Francisco, California), were reported to be compatible with detergents.^10^ The CPM dye assay is limited to proteins with buried cysteine residues, and the nature of the signal was questioned in literature.^21^ Finally, fluorescent dyes that respond to changes in viscosity were successfully used to report protein unfolding.^22^

Given the limitations outlined above, we chose measurements of intrinsic tryptophan (Trp) fluorescence in the UV range to obtain the best combination of sample consumption, reagent compatibility and data quality. This approach uses Trp residues as a built‐in probe to report protein unfolding: in a hydrophobic environment Trp UV fluorescence peaks around 330 nm, and in a hydrophilic the fluorescence peak is around 350 nm. During unfolding a Trp residue would typically move from the hydrophobic core of the protein to the solvent, thus producing a red‐shift in Trp fluorescence spectrum. A number of approaches to perform data reduction from a full spectrum to a single value was proposed^12^: (a) focusing on a single characteristic wavelength (e.g., fluorescence emission at 330 or 350 nm); (b) the difference between characteristic wavelengths; (c) ratio of characteristic wavelengths; (d) area under curve for the spectrum between specified wavelengths; (e) determination of the wavelength of maximum fluorescence intensity.

We chose Trp fluorescence measurements as the main readout to screen protein thermal stability for several reasons. First, the nature of the signal is well‐established and described.^23^ Second, the signal readout is compatible with most common buffer components, most notably detergents. Third, protein sequences usually contain at least one Trp residue (1% estimated probability^24^), and also other aromatic residues, for example, tyrosine (Tyr, 3% estimated probability^24^), can report unfolding.^25^, ^26^ Finally, recent developments in instrumentation (NanoDSF assay^11^, ^12^) provide high‐resolution (temperature control accuracy 0.1°C) thermal unfolding curves for up to 48 samples in parallel with as low as 0.5–1 μg protein per sample. To speed up data acquisition, the fluorescence intensity is only measured at two wavelengths (330 and 350 nm with excitation at 280 nm, F330 and F350) instead of the full spectrum. Fluorescence ratio at 350 and 330 nm (F350/F330 ratio) is then used as a proxy for global changes in the Trp fluorescence spectrum. Protein samples have to be loaded into glass capillaries, however, in our experience, this does not limit the throughput and all 48 samples can be transferred to the device within 10 min.

It should be noted that any bulk spectroscopic measurement provides only a partial description of the whole protein molecule. Indeed, CD measurements report changes in the secondary structure, while Trp fluorescence reports alterations in the hydrophobic core. Thus, the parameters derived from curve analysis will characterize the respective properties of a “melting unit”,^27^ that is, the molecular entity that elicits the observed signal. For the purposes of thermostablity screening it can be assumed that overall protein stability positively correlates with the stability of a melting unit. The apparent nature of the values is indicated with a prime sign (′) throughout the manuscript.

## 2 RESULTS

### 2.1 *Ranking measures derived from common protein unfolding models*

NanoDSF assay provides high resolution thermal unfolding curves with excellent throughput. However, the analysis implemented in the manufacturer's software (PR.ThermControl, NanoTemper GmbH, Munich, Germany) is limited to estimation of T_m_ and T_onset_ with a nondisclosed algorithm, which is presumably based on the analysis of the smoothened first derivative curve. In this section we use three models of protein unfolding (equilibrium thermodynamic, kinetic and empiric) to introduce measures of protein stability that capture all information available in the experimental data.

Protein unfolding is a complex multi‐step process. Recent studies of bacteriorhodopsin, a small α‐helical membrane protein, revealed multiple intermediates of different properties and lifetime.^28^, ^29^ A bulk spectroscopic measurement, as usually done in a thermal or chemical unfolding experiment, cannot capture this complexity, and the signal originating from the intermediate states is blended together.^30^, ^31^ Unless there is a long‐lived stable intermediate, and the transitions between states are widely separated, the unfolding curve would in many cases adopt a simple sigmoidal shape even for complex multimolecular assemblies. Thus, the problem of ranking the results of thermal unfolding screens can be often reduced to ranking of sigmoidal curves.

#### 2.1.1 *Equilibrium thermodynamic model*

Quantitative description of protein unfolding using classical thermodynamics is common and well‐established.^32^, ^33^, ^34^, ^35^, ^36^ Core thermodynamic characteristics of a protein, including enthalpy of unfolding at T_m_ (ΔH_m_), heat capacity change of unfolding (ΔC_p_) and Gibbs free energy of unfolding (ΔG_u_) can be obtained by thermal or chemical unfolding assays. The dependence of ΔG_u_ on temperature (T) is usually expressed as a variation of the Gibbs‐Helmholtz equation:

$$\Delta G_{u}\left(T\right)=\Delta H_{m}\cdot \left(1-\frac{T}{T_{m}}\right)+\Delta C_{p}\cdot \left(T-T_{m}-T\cdot \ln\frac{T}{T_{m}}\right)$$

ΔG_u_ is a particularly valuable measure, because it quantifies how thermodynamically favorable the folded state of the protein is under given conditions with higher ΔG_u_ corresponding to higher stability. ΔG_u_ calculations thus provide a natural way to combine the slope (ΔH_m_) and inflection point (T_m_) of a sigmoidal thermal unfolding curve into a single quantitative metric to rank the results. To ensure consistency with other sources of thermodynamic values,^37^ which typically refer to some standardized conditions, we propose to use ΔG_u_ extrapolated to 298.15 K and standard pressure (100 kPa), that is, ΔG_u_°.

#### 2.1.2 *Kinetic model*

Classical thermodynamic model of protein unfolding assumes that protein unfolding is a fully reversible reaction, and the equilibrium is reached at every step of a thermal unfolding experiment. In practice, proteins rarely exhibit such behavior, and a kinetic description of protein unfolding was proposed.^38^, ^39^ In the simplest case of irreversible unfolding from native state to unfolded (N → U), the fraction of state N (x_N_) as a function of temperature is described as:

$$x_{N}\left(T\right)=\int_{T_{\min}}^{T_{\max}}\frac{-1}{v}\cdot \exp\left(\frac{-E_{a}}{R}\cdot \left(\frac{1}{T}-\frac{1}{T_{f}}\right)\right)\cdot x_{N}$$

where T_min_ and T_max_ are the start and end temperatures of the measurement, v is the scan rate (degrees/min), E_a_ is the activation energy of unfolding, T_f_ is the temperature where reaction rate constant of unfolding (k_u_) equals 1, R is the universal gas constant. x_N_ is assumed to be 1 at T_min_, that is, the protein is fully folded in the beginning of the measurement. Similar to the classical thermodynamic model described above, T_f_ and E_a_ are the core characteristics of the sigmoidal unfolding curve: T_f_ describes the overall positioning of the curve on the temperature axis, while E_a_ corresponds to the slope of the curve. The key difference is that depending on the scan rate T_f_ may or may not coincide with the inflection point of the sigmoidal curve (maximum of the first derivative). Since the rate constant of unfolding is inversely related to protein stability, we propose to use the negative logarithm of k_u_ extrapolated to 298.15 K (pk_u_°) to rank the results of a thermal unfolding experiment described with this model:

$$pk_{u}{}^{\circ}=-\text{log}\;\text{exp}\left(\frac{-E_{a}}{R}\cdot \left(\frac{1}{298.15}-\frac{1}{T_{f}}\right)\right)$$

#### 2.1.3 *Empirical model*

The transition in thermal unfolding curves can be also described empirically by two characteristic temperatures: T_m_ (i.e., the mid‐point of the transition) and onset temperature T_onset_, which corresponds to the first detectable deviation of the experimental curve from the extrapolated linear baseline.^40^, ^41^ Since T_m_ and T_onset_ are orthogonal characteristics of the curve, they can be combined into a single measure by computing the Euclidean distance from point with 0 K coordinates in a scatter plot of T_m_ and T_onset_:

$$T_{\text{eucl}}=\sqrt{T_{m}^{2}+T_{\text{onset}}^{2}}$$

In this case the sample that has the most optimal combination of both T_m_ and T_onset_ will have the highest T_eucl_.

#### 2.1.4 *Models of protein unfolding exhibit high rank‐order correlation*

We tested if there are differences in protein stability ranking between the three measures described above. We used a set of diverse thermal unfolding curves of the *E. coli* multidrug transporter MdfA^42^ in a variety of detergents^40^ (see also “Protein stability heatmaps” section below). The curves were fit to a classic thermodynamic, kinetic and empirical model using MoltenProt software (see next section), and the final ranking parameters ΔG_u_°, pk_u_°, and T_eucl_ were calculated (Figure S1a). The pair‐wise scatter plots demonstrate strong linear correlation (Pearson correlation coefficient over 0.99) and suggest high rank order consistency (Kendall's τ in range 0.91–0.98). Thus, if the signal can be described with a sigmoidal curve, the same conditions will come up as the top hits in a thermal unfolding screen regardless of the choice of the protein unfolding model. To avoid redundancy, we will use ΔG_u_° throughout the rest of the manuscript.

#### 2.1.5 *Linear extrapolation of ΔG_u_ is sufficient for relative comparison*

According to the Gibbs‐Helmholtz equation, three thermodynamic parameters are required to calculate ΔG_u_ at a specific temperature. ΔH_m_ and T_m_ can be readily obtained from thermal unfolding assays,^26^ while experimental estimation of ΔC_p_ usually requires multiple DSC runs and is not always feasible. ΔC_p_ primarily depends on the size of the protein,^43^ and as the first approximation ΔC_p_ can be assumed independent of temperature, pH or buffer composition.^32^ Neglecting the contribution of ΔC_p_ to ΔG_u_ will introduce a systematic error to ΔG_u_ with the following dependence on T_m_ and reference temperature T_ref_:

$$\text{Error}\left(T_{\mathrm{ref}},T_{m}\right)=\Delta C_{p}\cdot \left(T_{\mathrm{ref}}-T_{m}-T_{\mathrm{ref}}\cdot \ln\frac{T_{\mathrm{ref}}}{T_{m}}\right)$$

Since T_ref_ < T_m_, this error is always negative. Thus, assuming zero ΔC_p_ would produce an overestimate of ΔG_u_, as demonstrated with chicken egg lysozyme in the next section. In a typical thermal unfolding screen one is not concerned with absolute values of protein stability, but rather in the relative ranking to identify the most stabilizing conditions. ΔΔG_u_ between a sample and a reference sample (e.g., wild‐type protein, or original buffer condition) computed with ΔC_p_ being neglected will be close to the real ΔΔG_u_ as long as the difference in T_m_ is within 10–15 K.^32^ Unless otherwise stated, ΔC_p_ was not taken into account for ΔG_u_° calculations.

### 2.2 *Thermodynamic values obtained with MoltenProt are reliable and agree with orthogonal assays*

Application of the concepts outlined in the previous section to a high‐throughput assay such as NanoDSF is challenging, because it requires a robust nonlinear curve fitting procedure and an easy way to view the results. This prompted us to develop a software package MoltenProt^44^ (Figure 1(c), Figure S1c), which we successfully used to characterize a large NanoDSF dataset.^40^ Our previous work, however, did not compare the thermodynamic parameters obtained by the combination of NanoDSF and MoltenProt with other methods. To address this, we measured thermal unfolding profiles of model proteins (chicken egg lysozyme and ribonuclease A [RNase] from bovine pancreas) in the presence of increasing concentrations of chemical denaturants (urea and guanidine hydrochloride [GuHCl]). ΔH_m_ at zero denaturant concentration agreed well with literature data^45^, ^46^ (Table S2). To further validate the result, we computed ΔC_p_ by performing a linear fit of the ΔH_m_(T_m_) dependence (Figure S2a) and found good agreement of obtained ΔC_p_ values with literature data^45^, ^46^ (Table S2). Furthermore, in case of lysozyme we could obtain an independent estimate of ΔG_u_°′ by fitting the values of F350/F330 ratio at 298 K at different denaturant concentrations (Figure S2b, Table S2). As expected, ΔG_u_°′ (ΔC_p_ assumed zero) obtained from thermal unfolding was 17.9 kJ/mol higher than ΔG_u_°′ from chemical unfolding. If ΔC_p_ was taken into consideration, then the ΔG_u_°′ values agreed well (Table S2). These results demonstrate high consistency between thermal and chemical unfolding properties of model proteins when characterized with NanoDSF technique. A similar result was obtained previously for R16 α‐spectrin domain.^11^

We next analyzed thermal unfolding of human filamin C domain 19 (FlnC‐d19, see also below) with DSC, CD, and Trp fluorescence (Figure S2c, S2d, Table S3). T_m_ and ΔH_m_ values agreed well for all assays. ΔC_p_ from Trp fluorescence data (4,846 J/mol/K) is similar to the DSC result (4,910 J/mol/K) and was lower than the theoretical value (5,742 J/mol/K, see Table S3).

Finally, we assessed the overall level of uncertainty in ΔG_u_°′ estimation, which is derived from combined errors in ΔH_m_ and T_m_. We measured several protein samples in replicates on different days (Table S4). Typically, standard deviation of the measurement was in the range of 0.3–0.9 K for T_m_ (below 0.5% relative error) and 10–50 kJ/mol for ΔH_m_ (typically, 3–10% relative error). This result highlights the high quality of the data obtained with Trp fluorescence. Previous work^7^ reported up to 30% relative error in ΔH_m_ estimation and 0.5 K error for T_m_ obtained with the modified Thermofluor assay.

### 2.3 *Case studies*

#### 2.3.1 *Ranking based exclusively on T_m_ may produce misleading results*

**FIGURE 2:**
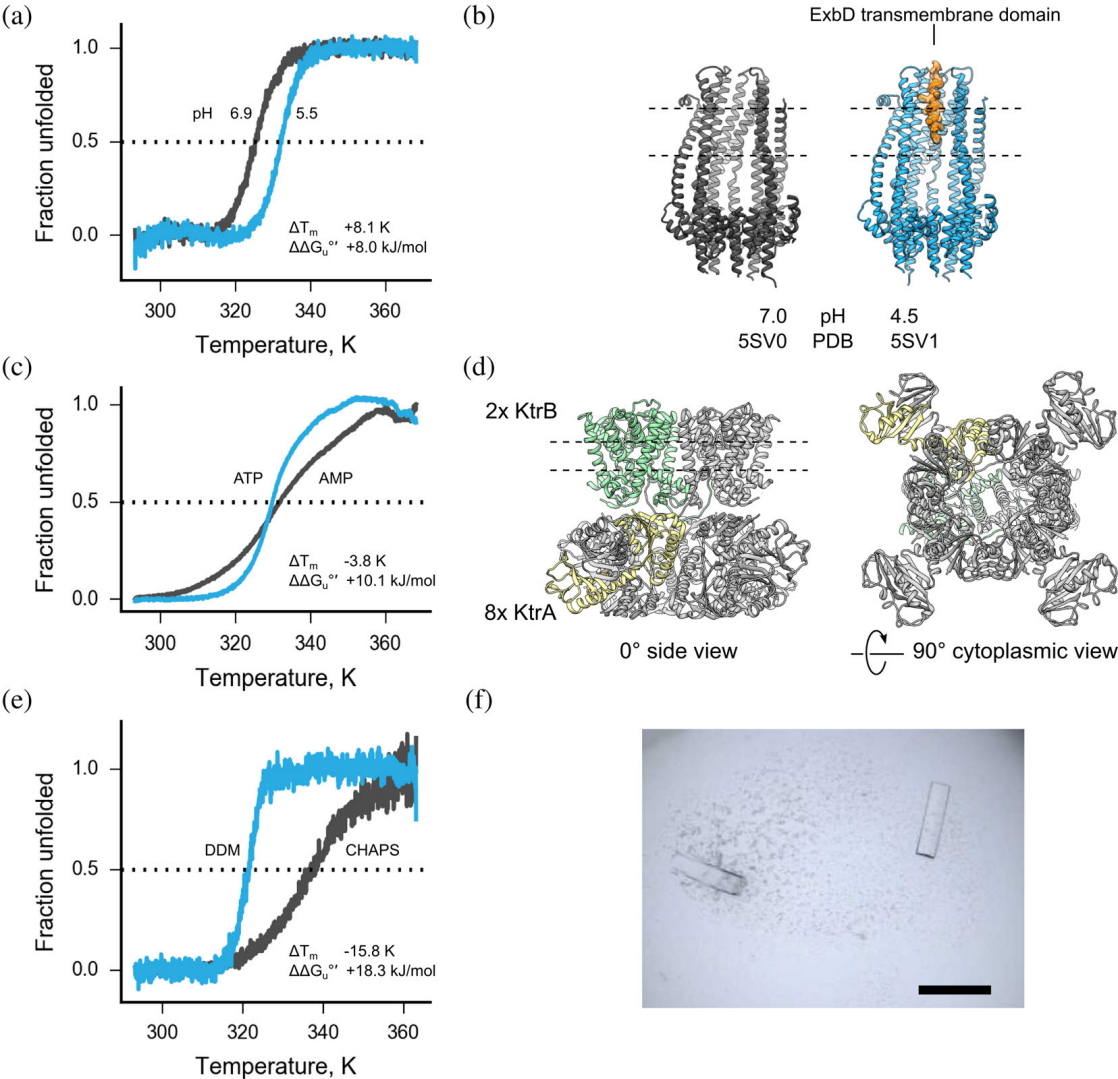
ΔG_u_°′ is a superior thermostability metric compared to T_m._ (a) Representative thermal unfolding curves of ExbBD complex at pH 5.5 (blue) or pH 6.9 (gray). Raw fluorescence readings are provided in Figure S2e. (b) Crystal structure of ExbBD at pH 4.5 (blue) and pH 7.0 (gray). The transmembrane domain of ExbD (orange) is only visible in crystals formed at pH 4.5, which have higher thermostability. Two ExbB subunits were removed from display to visualize the lumen of the channel. (c) Representative thermal unfolding curves of KtrAB complex in presence of ATP (blue) or AMP (gray). Raw fluorescence readings are provided in Figure S2f. (d) Crystal structure of KtrAB in complex with ATP (PDB 4J7C). Two KtrB subunits are buried in the membrane and perform cation transport; nucleotide‐bound KtrA forms a tetramer of dimers. The approximate position of the lipid bilayer is shown with dashed lines. One KtrB subunit is shown in green and one KtrA subunit is shown in yellow. (e) Representative thermal unfolding curves of DgoT in presence of DDM (blue) or CHAPS (gray). Raw fluorescence readings are provided in Figure S2g. f) Crystals of the transporter DgoT in purified in 0.03% DDM and crystallized in 0.03 M MgCl_2_, 0.1 M MES pH 6.5, 28% polyethyleneglycol‐400 (PEG‐400). Scale bar 200 μm. In thermal unfolding curves the intersection with the horizontal dashed line at 0.5 fraction unfolded corresponds to T_m_. ΔT_m_ and ΔΔG_u_ are computed by subtraction of the value for a less stable state (gray unfolding curve) from the value of a more stable state (blue unfolding curve). Specific values as well as information on the replicates are provided in Table S5

We used MoltenProt to characterize thermal unfolding of a variety of proteins and protein complexes (Figure 1(d)). In many cases, the result ranking with either ΔG_u_°′ or T_m_ gave similar results. For instance, the ExbBD complex^47^ (Figure 2(a), Table S5) had a higher T_m_ and ΔG_u_°′ at pH 5.5 compared to pH 6.9. In a number of cases, however, focusing on T_m_ was misleading.

KtrAB is a nonselective prokaryotic cation channel composed of a ring of four KtrA dimers that bind adenosine nucleotides (ATP, ADP, and AMP) and two KtrB subunits embedded in the membrane forming the channel^48^ (Figure 2(d)). We analyzed thermostabilty of KtrAB in presence of two known ligands, ATP and AMP. While T_m_ was similar for both samples, ΔG_u_°′ decreased two‐fold in presence of AMP (Figure 2(c), Table S5). This agrees with biochemical data (functional activation with ATP, low affinity for AMP) and structural research (ATP‐bound state of the complex produced crystals diffracting to 3.5 Å^48^).

DgoT, a prokaryotic sugar transporter, was initially purified in the detergent DDM (0.03% final concentration),^49^ and then the detergent was exchanged to 1.2% CHAPS. We observed a 15.6 K increase in T_m_ (from 321.3 K in DDM to 337.1 K in CHAPS), whereas ΔG_u_°′ decreased two‐fold (Figure 2(e), Table S5). The increase in T_m_ is somewhat unexpected, because CHAPS is a zwitter‐ionic detergent, and this type of detergents is generally more harsh compared to nonionic ones, including DDM.^50^ Furthermore, the protein could only be crystallized in maltoside detergents (DM, UDM, DDM, Figure 2(f)).

Taken together, these results indicate that T_m_ must be used with great care when selecting hits in protein thermal unfolding screens. Such screens are usually performed at early stages of a project, so following up a false‐positive hit can result in significant loss of time and resources. This can be easily avoided by combining inflection point and slope of the sigmoidal curve in a simple quantitative measure, such as ΔG_u_°′.

#### 2.3.2 *Functional insights obtained from ΔG_u_°′*

Measuring protein characteristics obtained with thermal unfolding assays can provide useful insights about their functional interactions. ExbBD is part of the Ton complex embedded in inner membranes of Gram‐negative bacteria and plays an important role in nutrient uptake.^47^ Conductivity measurements indicated that the complex is fully functional at pH above 6.5, while lower pH values decrease conductivity.^47^ We compared thermostability of ExbBD at different pH values using 100 mM broad‐range buffer system SPG (succinate/phosphate/glycine mixed in molar ratio 1:4:3), and observed higher stability at lower pH values (Figure 2(a), Table S5). This indicates that the closed state of ExbBD is thermodynamically more stable, which might be attributed to the stabilization of the ExbD transmembrane domains in the channel formed by the ExbB pentamer (Figure 2(b)).

**FIGURE 3:**
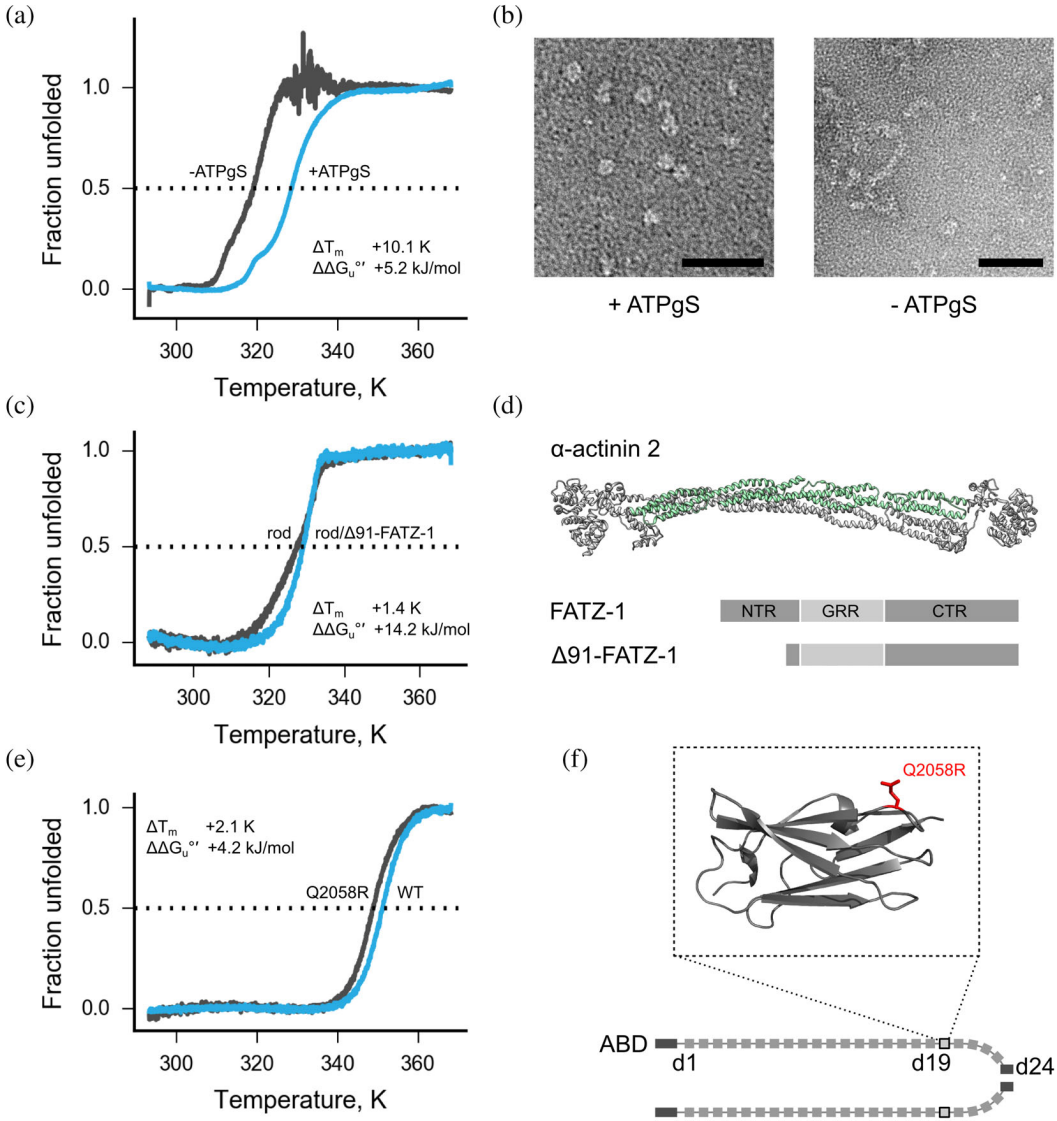
Functional insights obtained by ΔG_u_°′ analysis. (a) Representative thermal unfolding curves of RuvB in presence or absence of ATPgS (blue and gray). Raw fluorescence readings are provided in Figure S3a. (b) RuvB in presence or absence of ATPgS visualized with negative staining electron microscopy (representative images, scale bar 50 nm). Full‐size micrographs are provided in Figure S3b. (c) Representative thermal unfolding curves of α‐actinin‐2 rod domain (gray) and its complex with the intrinsically disordered protein Δ91‐FATZ‐1 (blue). Raw fluorescence readings are provided in Figure S3c. (d) Crystal structure of α‐actinin‐2 dimer (PDB 4D1E) and a schematic representation of FATZ‐1. Rod domain (residues 274–746) of one α‐actinin‐2 subunits is shown in green. Δ91‐FATZ‐1 construct contains a deletion of 91 N‐terminal residues. NTR, N‐terminal region; CTR, C‐terminal region; GRR, glycine‐rich region. (e) Representative thermal unfolding curves of wild type FlnC‐d19 (blue) and cardiomyopathy causing mutation Q2058R (gray). Raw fluorescence readings are provided in Figure S3e. (f) Schematic diagram of FlnC and a model of FlnC‐19 bearing a cardiomyopathy‐causing mutation Q2058R (inset, residues 2036 to 2,130; Q2058R is highlighted in red). The model is based on a partially refined crystal structure of FlnC domains 18 and 19 (Mlynek et al., manuscript in preparation). In thermal unfolding curves (a, c, e) the intersection with the horizontal dashed line at 0.5 fraction unfolded corresponds to T_m_. ΔT_m_and ΔΔG_u_are computed by subtraction of the value for a less stable state (gray unfolding curve) from the value of a more stable state (blue unfolding curve). Specific values as well as information on the replicates are provided in Table S5

RuvB is an ATPase that resolves the Holiday junction.^51^, ^52^ Similar to many ATPases, it assembles to a functional hexamer upon addition of ATP or its analogs. We compared thermostability of RuvB from *Streptococcus thermophilus* in presence and absence of 1 mM adenosine 5′‐(γ‐thio)‐triphosphate (ATPgS), a slowly hydrolysable ATP analog. The enzyme was heavily destabilized without its ligand and produced a weak signal in the thermal unfolding assay, whereas a ligand‐bound state was more stable (Figure 3(a)). These results agree well with electron microscopy analysis of the sample, where ATPgS‐free protein showed mainly aggregated particles, while ATPgS‐bound RuvB demonstrated a homogeneous population of ring‐like particles (Figure 3(b)).

FATZ‐1 (calsarcin‐2/myozenin‐1) is an intrinsically‐disordered protein found in the Z‐discs of skeletal muscles, the boundaries between two adjacent sarcomeres. FATZ‐1 binds to the major Z‐disc protein α‐actinin‐2, which functions as a homodimer.^53^, ^54^, ^55^, ^56^ We generated a complex of FATZ‐1 lacking the first 91 residues (Δ91‐FATZ‐1) with the rod domain of α‐actinin‐2 (Figure 3(d)). The complex consists of two Δ91‐FATZ‐1 molecules bound to one rod domain dimer, as demonstrated by size‐exclusion chromatography coupled with multi‐angle laser light scattering (SEC‐MALLS, Figure S3d). In thermal unfolding assays ΔG_u_°′ of the complex was almost two‐fold higher than ΔG_u_°′ of the rod domain alone (Figure 3(c), Table S5) confirming the functional interaction between the proteins.

Filamin C (FlnC) is located at premyofibrils, myofibrillar Z‐discs and myofibrillar attachment sites of striated muscle cells, where it is involved in mechanical stabilization, mechanosensation and intracellular signaling.^57^, ^58^, ^59^ FlnC is a homodimer, where each subunit consists of an N‐terminal actin‐binding domain (ABD) followed by 24 immunoglobulin‐like (Ig‐like) domains (Figure 3(f)). Mutations in FlnC give rise to skeletal muscle diseases and cardiomyopathies.^60^ We investigated stability and fold integrity of FlnC mutant Q2058R implicated in cardiomyopathy located in the Ig‐like domain 19 of FlnC (FlnC‐d19, Figure 3(f), inset). ΔG_u_°′ of wild‐type FlnC‐d19 was 53.0 kJ/mol, while in the case of the Q2058R mutant it decreased by 4.1 kJ/mol, suggesting that a single mutation can destabilize FlnC‐d19.

#### 2.3.3 *Protein stability heatmaps*

**FIGURE 4:**
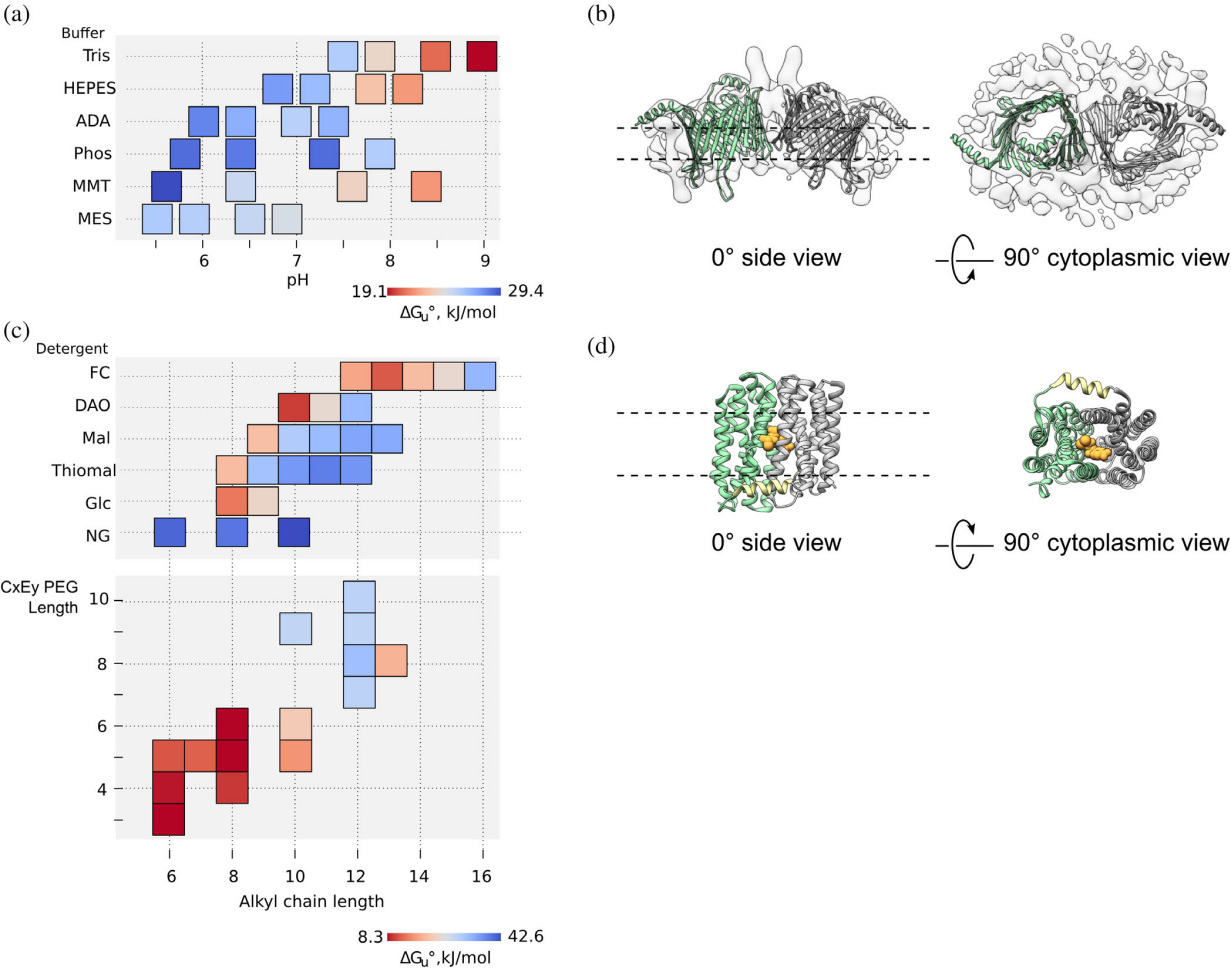
Exemplary ΔG_u_°′ heatmaps. (a) Stability of the TOM core complex as a function of pH and buffer system. Each square in the heatmap represents an experimental measurement of ΔG_u_°′ (*n* = 2). The middle point of each square is positioned on either categorical axis (buffer system) or numeric axis (pH). Indicated buffer system was added to a final concentration of 100 mM to the original buffer system (20 mM HEPES, pH 7.2). (b) Structure of the TOM core complex. Two copies of Tom40 model (PDB 5O8O, green and gray) were placed in the 6.8 Å electron density map (EMDB 3761). The approximate position of the lipid bilayer is shown with dashed lines. (c) Stability of MdfA as a function of detergent chemistry. Each square in a heatmap represents an experimental measurement of ΔG_u_°′ (n = 2). The middle point of each square is positioned on either categorical axis (detergent family) or numeric axis (alkyl chain length). Lower heatmap shows the heatmap for alkyl ether detergents (CxEy), where the length of alkyl chain (Cx) is on X‐axis and the length of the hydrophilic PEG component (Ey) is on Y‐axis. FC, fos‐choline; DAO, dimethylamineoxide; Mal, maltoside; Thiomal, thiomaltoside; Glc, glucoside; NG, maltose neopentyl‐glycol. (d) Crystal structure of MdfA in complex with chloramphenicol (PDB 4ZOW). The protein contains two pseudo‐symmetric domains (green and gray) and the amphipatic interdomain linker (yellow). Bound chloramphenicol is shown in orange; the approximate position of the lipid bilayer is shown with dashed lines

Minimal sample consumption by modern equipment for thermal unfolding measurements combined with MoltenProt analysis is an excellent tool for sampling and visualizing protein stability as a function of various conditions. Figure 4(a) represents a heatmap view of stability of the TOM core complex, a protein precursor entry gate in eukaryotic mitochondria,^61^ in a variety of buffer systems and pH values. The optimal pH value for the TOM core complex was specific to the buffer system used. Remarkably, the stability of the complex was independent of pH in phosphate buffer, which could be attributed to the salting‐in effect of phosphate ions.^62^, ^63^ In most other buffer systems the optimal pH range for the TOM core complex is narrow with an overall trend towards moderately acidic pH values.

Due to the broad reagent compatibility, Trp fluorescence is widely used to assess stability of membrane proteins.^49^, ^64^, ^65^ Figure 4(c) presents the ΔG_u_°′ heatmap of MdfA in a variety of detergents. We identified two important trends: (a) in agreement with a broad body of evidence,^66^ detergents with longer alkyl chain lengths increase the stability of MdfA within a single detergent family; (b) detergent headgroup chemistry defines the stabilizing properties of the detergent. In particular, neopentyl‐glycol detergents,^67^, ^68^ albeit with relatively short alkyl chains, promote stability of MdfA, whereas zwitter‐ionic fos‐choline detergents are harsh towards the protein regardless of the chain length. We observed a similar trend by testing additional eight membrane proteins.^40^ This result suggests that in order to identify a suitable detergent for a particular protein it is sufficient to screen a limited number of common detergent families and then optimize the alkyl chain length within the best‐ranking detergent family. If detergents with shorter alkyl chains are not available, organic solvents known to decrease micelle size (e.g., ethanol or heptanetriol^69^) can be tested.

#### 2.3.4 *Signal detection in lieu of Trp residues*

Trp belongs to one of the most rare residues found in protein sequences.^24^ Furthermore, only the Trp residues that change their environment (hydrophobic to hydrophilic, but also vice versa) over the course of an unfolding experiment will provide a usable signal. Tyr and phenylalanine (Phe) residues are also capable of reporting protein unfolding, as demonstrated previously^25^, ^26^ and also in this work with RNase (Tables S2 and S5). To gain further insights in the assay requirements we measured thermal unfolding of ubiquitin (Table S5), which contains only two Phe and one Tyr residue. The protein had to be used in concentrations above 0.5 mg/ml in order to obtain sufficient fluorescence counts. In agreement with the published data,^70^ the thermostability of ubiquitin was very high. The full unfolding curve could not be recorded in the available temperature range of the device (20–95°C). Due to the absence of the posttransition baseline T_m_ was estimated from the peak of the derivative curve and was 365 K. Thus, even a protein with very few fluorescent residues can be characterized with NanoDSF. It should be noted, however, that according to the Fourier transform infrared spectroscopy analysis of ubiquitin unfolding, the molecule does not undergo significant unfolding at the temperatures above 85°C, but rather forms aggregates via intermolecular β‐sheets.^70^

## 3 DISCUSSION

The ultimate objective of a thermal unfolding screen is to boil down a large set of conditions (typically, 100–300) to a smaller set for in‐depth characterization with orthogonal low‐throughput techniques. Thus, in many cases the simplicity of analysis is preferred over accuracy, and T_m_ is so far the most widely used readout for thermal unfolding assays. While being a reliable indicator of protein stability with proved empirical performance, T_m_ only partially describes the unfolding process, and a situation where T_m_‐based ranking produces a misleading result can be easily modeled.

In this work we introduce an improved framework for quantitative characterization of protein stability from thermal unfolding assays. We derive new measures from classic thermodynamic, kinetic and empirical models of protein unfolding that incorporate all characteristics of a sigmoidal curve (inflection point and slope). We show that these measures can be used interchangeably and help avoid the pitfalls that are possible when samples are ranked exclusively with melting temperature T_m_.

Application of outlined concepts into practice, particularly, with the purpose of high‐throughput analysis, may be challenging in terms of data analysis and management. To aid this task we designed an open source package MoltenProt, which aims to minimize human intervention and present the user with a minimal set of decision points. Currently MoltenProt supports Trp fluorescence data from Prometheus NT.48 as well as plain text formats (e.g., for analysis of CD data). The open source nature of the code allows easy tailoring and extension of MoltenProt for custom applications, for example, new types of readouts. We believe that analysis of label‐free thermal and chemical unfolding data with MoltenProt will help biochemists, biophysicists and structural biologists to systematically assess the stability of their samples and serve as a guide to better structures and faster results. Potential applications include buffer and construct optimization for structural and biochemical studies as well as validation of existing buffer formulations, screening for small‐molecule binding partners and comparison of effects of point mutations.

## 4 MATERIALS AND METHODS

### 4.1 *Protein samples*

Chicken egg lysozyme and RNase A were purchased from Sigma‐Aldrich (Saint Lois, Missouri). Lysozyme was dissolved in 50 mM sodium phosphate buffer, pH 7.5, 150 mM NaCl at concentration 20 mg/ml. RNase A was dissolved in 10 mM Tris–HCl, pH 7.5, 15 mM NaCl at concentration 20 mg/ml.

DgoT was expressed and purified as described previously.^49^ Protein crystals formed at DgoT concentration 5 mg/ml in 0.03 M MgCl_2_, 0.1 M MES, pH 6.5, 28% v/v PEG‐400 using the vapor diffusion technique (ratio 1:1) at 20°C.

Purification and detergent screening of MdfA was described previously.^40^

ExbBD was purified as described previously.^47^ The buffer system/pH were changed by adding a new buffer system to final concentration 100 mM.

KtrAB was purified and assembled as described previously.^48^ The nucleotides were added to KtrA at the affinity chromatography step (5 mM ATP or 50 mM AMP). SEC of KtrA was performed in absence of any nucleotides; pooled fractions were supplemented with either 1 mM ATP or 10 μM AMP and dialyzed against 50 mM Tris–HCl, pH 7.5, 150 mM KCl, 5 mM DTT with either 1 mM ATP or 10 μM AMP. KtrA‐ATP or KtrA‐AMP was added to KtrB at the affinity chromatography step of KtrB purification in buffer supplemented with 1 mM ATP or 10 μM AMP. The complex was dialyzed and further purified by SEC in 20 mM Tris–HCl, pH 8.0, 120 mM NaCl, 30 mM KCl, 5 mM DTT, 1.5 mM CYMAL‐6 without any nucleotide added.

Coding sequence of RuvB from *S. thermophilus* was cloned into pET‐52b(+) vector and purified using nickel affinity chromatography followed by tag cleavage with tobacco etch virus protease and size‐exclusion chromatography at Superdex‐200 column (GE Healthcare, Chicago, Illinois) equilibrated with 100 mM Tris–HCl, pH 8.0, 100 mM NaCl, 1 mM ethylenediaminetetraacetic acid (EDTA), 0.5 mM DTT, 15% w/v glycerol.

TOM core complex was purified as described previously.^61^ The pH/buffer system screen was prepared in‐house (stock concentration 0.5 M). Three microliter of the screen were added to 12 microliter of 0.25 mg/ml TOM core complex in 20 mM HEPES, pH 7.2, 2% v/v DMSO, 350 mM KCl, 0.1% w/v DDM. In addition, a triplicate of the sample in the original buffer was measured to assess the uncertainty in curve parameter estimation and a water control to assess the destabilizing effect of dilution, where an equal volume of ultra‐pure water was added instead of the screen.

FlnC‐d19, Δ91‐FATZ‐1, and α‐actinin‐2 rod were prepared using the platform and protocols described previously.^71^ The detailed purification protocols for individual samples will be published elsewhere (Mlynek et al., manuscript in preparation). Protein concentration was determined from absorbance at 280 nm. Where applicable, extinction coefficients were calculated from the primary amino acid sequence using ProtParam.^72^

Ubiquitin with a C‐terminal His‐tag (sequence MASMTGGQQMGRGSMQIFVKTLTGKTITLEVEPSDTIENVKAKIQDKEGIPPDQQRLIFAGKQLEDGRTLSDYNIQKESTLHLVLRLRGGKLAAALEHHHHHH) was cloned in pET21a vector and purified in 10 mM sodium/potassium phosphate buffer, 137 mM NaCl, 2.7 mM KCl using Ni‐affinity chromatography followed by size‐exclusion chromatography on Superdex‐75 column (GE Healthcare, Chicago, Illinois).

### 4.2 *Trp fluorescence measurements (NanoDSF assay)*

Prometheus NT.48 (NanoTemper GmbH, Munich, Germany) was used to run all Trp fluorescence measurements. Standard‐grade glass capillaries were filled with 10–15 μl of the sample, excitation light was preadjusted to get fluorescence readings above 2000 arbitrary units for F330 and F350, and samples were measured in temperature range 20–95°C or 15–95°C with temperature slope of 1°C/min. Up to 48 samples could be measured simultaneously. Prior to the measurements samples were centrifuged for 10 min at 16000*g* at 4°C to remove any large aggregates.

### 4.3 *CD spectroscopy*

Electronic circular dichroism was measured using Chirascan (Applied Photophysics, Leatherhead, UK). Prior to the measurements the xenon arc lamp, monochromator and sample chamber were flushed with nitrogen. Temperature was increased stepwise in range 20–95°C with slope of 1°C/min using a Peltier element. UV–vis absorbance and CD were measured at path length 0.5 mm, with 1 nm spectral bandwidth and 7 s scan time. FlnC‐d19 was measured at concentration 0.59 mg/ml in 20 mM phosphate buffer, 150 mM sodium fluoride, pH 7.4.

CD at 208 and 218 nm as a function of temperature was processed in MoltenProt as described below.

### 4.4 *DSC*

To achieve identical buffer composition for reference and sample cells every sample was buffer exchanged using a Superdex‐200 5/150 GL or Superdex‐200 10/300 Increase column (GE Healthcare, Chicago, Illinois) connected to an HPLC 1260 Infinity with fraction collector (Agilent Technologies, Santa Clara, CA).

DSC experiments were performed with the PEAQ Differential Scanning Calorimeter Automated (Malvern Panalytical, Malvern, UK). Experiments were performed under increased pressure (~62 psi) to prevent the solutions from boiling. At the beginning of an experiment, at least four buffer runs (i.e., buffer in both sample and reference cells) were performed to establish the thermal history of the cells and to collect optimal buffer scans for buffer subtraction.

A temperature slope of 1°C/min was used for all experiments. Upon scanning from 20°C to 130°C and reheating again, we did not encounter any protein refolding. Therefore, the reheated run (i.e., rescan) was taken for buffer subtraction. Data analysis was performed with the PEAQ‐DSC analysis software (Malvern Panalytical, Malvern, UK). The baseline was fitted with the spline baseline correction model to account for differences in the heat capacities of the folded and unfolded states of the protein. Concentration normalization was performed and transitions were fitted with a two‐state thermal unfolding model.

### 4.5 *Characterization of lysozyme and RNase a unfolding*

Eight molar stock of GuHCl was prepared by mixing 7.64 g of GuHCl with 4.21 ml assay buffer (50 mM sodium phosphate buffer, pH 7.5, 150 mM NaCl). pH of GuHCl solution was further adjusted to pH 7.5 using 1 M Tris, pH 8.0. Nine molar stock of urea was prepared freshly by mixing 5.41 g urea with 5.9 ml 1x assay buffer. One microliter of concentrated protein stock (final concentration 0.1 mg/ml for lysozyme and 0.5 mg/ml for RNase A) was added to 40 μl of series of denaturant concentrations and the mixture was incubated for 1 hr and 16 hr at 25°C.

Thermal unfolding data were processed in MoltenProt as described below to obtain ΔH_m_ and T_m_ values at different denaturant concentrations. Scatter plots of ΔH_m_ as a function of T_m_ demonstrate a clear linear dependence (Figure S2a). According to the definition of ΔC_p_:

$${\left(\frac{\mathrm{\delta \Delta H}}{\mathrm{\delta T}}\right)}_{p}=\Delta C_{p}$$

the slope of this line corresponds to the ΔC_p_ value.

Readings of fluorescence at the start of thermal unfolding measurement as a function of denaturant concentration were fit to the equation described by Santoro and Bolen^73^ to calculate m (changes in ΔG_u_ for each 1 M of denaturant) and ΔG_u_°′ at zero denaturant concentration.

Curve fitting was performed using scipy.curve_fit function.

### 4.6 *Electron microscopy*

Four microliter of RuvB in 20 mM Tris, pH 8.0, 20 mM NaCl, 10 mM MgCl_2_, 2% glycerol, 0.5 mM DTT with or without 1 mM ATPgS was applied onto home‐made glow‐discharged carbon‐coated Cu/Pd grids and incubated for 30 s. The grids were stained with 2% uranyl acetate for 30–40 s, blotted and visualized in Talos L120C (FEI Company, Hillsboro, Oregon) microscope using TEM Imaging and Analysis Software (FEI Company, version 4.15).

### 4.7 *SEC‐MALLS*

Analytical SEC was performed using HPLC 1260 Infinity (Agilent Technologies, Santa Clara, California), linked to a Superdex‐200 10/300 Increase column (GE Healthcare, Chicago, Illinois). Column was preequilibrated with buffer containing 20 mM Tris–HCl, pH 7.5, 100 mM NaCl, 50 mM arginine, 50 mM glutamic acid, 0.5 mM phenylmethylsulfonyl fluoride (PMSF), 1 mM EDTA, 1 mM β‐mercaptoethanol). Hundred microliter of sample was injected at concentration between 2 and 4 mg/ml and the separation was performed at 20°C using a flow rate of 0.5 ml/min.

Online MALLS detection was performed with a miniDawn Treos detector (Wyatt Technology, Santa Barbara, California) using a laser emitting at 690 nm. Protein concentration was measured by refractive index using a Shodex RI‐101 (Showa Denko, Tokyo, Japan) using a typical refractive index increment (dn/dc) of 0.186 ml/g.^74^ Average molecular weight was calculated with Astra software (Wyatt Technology, Santa Barbara, California).

### 4.8 *High‐throughput analysis of thermal unfolding data*

Thermal unfolding and aggregation curves were read by MoltenProt directly from spreadsheet files generated by Prometheus NT.48 control software (PR.ThermControl, NanoTemper GmbH, Munich, Germany). Derivatives of experimental curves were computed with Savitzky–Golay filter (as implemented in scipy.signal.savgol_filter) using 4‐th order polynomial and window size of 10 K. Derivative curves were only used to provide an initial estimate for T_m_.

In most cases Prometheus NT.48 produces high quality data, which can be used directly for curve fitting and analysis. For suboptimal curves MoltenProt provides preprocessing steps to “rescue” the data. These include removal of data points in the start or end of curves, curve smoothing with median filter and data binning to suppress noise and expose trends.

Nonlinear fitting requires estimation of starting values for all parameters, and good starting values increase the convergence of the algorithm and may also prevent reaching the wrong local minimum. To estimate starting values for pre‐ and posttransition baseline parameters MoltenProt runs linear fitting of 10 K stretches in the beginning and the end of the thermal unfolding curve. Initial values of the baselines are also used to determine if the curve is S‐shaped (signal increases with temperature) or Z‐shaped (signal decreases with temperature). This information is important for obtaining an initial value for T_m_: if the signal increases with temperature, then the temperature corresponding to the local maximum of the derivative is taken as an initial value. Alternatively, the local minimum of the derivative curve is used. Obtaining robust initial values for ΔH_m_ is not straightforward, so the initial value was always set to 100,000 J/mol.

Curve fitting routine supports setting parameter bounds (as part of Trust Region Reflective algorithm implemented in scipy.curve_fit). For T_m_ the bounds were set to the temperature range of the experiment. ΔH_m_ was restricted to 60–4,000 kJ/mol. The bounds for baseline parameters were not set.

Several values were used to characterize the fitting results and filter out suboptimal unfolding curves (Figure S1c). The quality of the fit was quantified with standard error of the estimate S:

$$S=\sqrt{\frac{\sum {\left(F_{\exp}-F_{\mathrm{fit}}\right)}^{2}}{N-n}}$$

Where F_exp_ is experimental signal at given datapoint, F_fit_ respective fit value at given datapoint, N is total number of datapoints and n is number of fit parameters.

The standard deviation of all fit parameters was computed as a square root of diagonal values from fit parameter covariance matrix reported by scipy.curve_fit function. The curves with standard deviation for T_m_ above 0.5 K were considered unreliable fits and were discarded.

Finally, to compare the height of unfolding transitions between different samples and readouts we introduced baseline separation factor (BS‐factor):

$$\mathrm{BS}-\text{factor}=1-\frac{6\cdot S}{\left|k_{U}\cdot \mathrm{Tm}+b_{U}-k_{N}\cdot T_{m}-b_{N}\right|}$$

Where S is standard error of estimate, k_N_, b_N_ – slope and intercept of the pretransition baseline (temperature dependence of native state fluorescence), k_U_ and b_U_ – slope and intercept of the posttransition baseline (temperature dependence of unfolded state fluorescence) as obtained from nonlinear curve‐fitting and T_m_ is melting temperature. BS‐factor metric relates the uncertainty of the fit, expressed as standard error of the estimate, and the distance between the pre‐ and posttransition baselines at T_m_ (Figure S1b) and is conceptually similar to Z‐factor commonly applied to assess quality of high‐throughput screens.^75^ If the baselines are too close to each other, then their variability bands overlap, and the unfolding transition cannot be well distinguished. BS‐factor is dimensionless and can be used to compare the quality of different readouts. In general, curves with BS‐factor from 0.5 to 1 are considered excellently separated, while negative BS‐factor indicates an unreliable curve.

For the majority of the data the F350/F330 ratio was used (Table S5). In some cases the BS‐factor for F350/F330 ratio data was suboptimal, and the fluorescence at individual fluorescence wavelength was used. Shape of unfolding curves is similar between F330 and F350 readouts, so only F330 was used for analysis.

For the purposes of visualization we computed the baseline‐corrected experimental curves, that is, the fraction unfolded versus temperature^73^:

$$K_{u}\left(T\right)=\frac{f_{\mathrm{unf}}}{f_{\text{native}}}=\frac{k_{N}\cdot T+b_{N}-\text{Signal}\left(T\right)}{\text{Signal}\left(T\right)-k_{U}\cdot T-b_{U}}$$

$$f_{\mathrm{unf}}=\frac{K_{u}\left(T\right)}{1+K_{u}\left(T\right)}=\frac{\text{Signal}\left(T\right)-k_{N}\cdot T-b_{N}}{k_{U}\cdot T+b_{U}-k_{N}\cdot T-b_{N}}$$

where f_unf_ is fraction of U state, f_native_ is fraction of N state, K_u_ is equilibrium constant of unfolding and other variables as described above.

The results of individual experiments can be visualized in an interactive HTML report or using a graphical user interface (GUI, Figure 1(c)). MoltenProt can export the results for further analysis and manipulation in a spreadsheet (XLSX) and comma‐separated value (CSV) format.

In addition to the data produced by PR.ThermControl, MoltenProt supports import of plain CSV files with one or more samples. CD data were converted to this format and analyzed as described above.

MoltenProt is written in Python v.3.8 using the following modules: pandas,^76^ scipy,^77^ numpy,^78^ matplotlib,^79^ PyQt5 (Riverbank Computing).

### 4.9 *Code availability*

MoltenProt is open source/libre software licensed under GNU General Public License (version 3 or above). The source code is supplied in the Supplementary material and also available via http://www.marlovitslab.org.

### 4.10 *Additional software*

Molecular visualization was performed with UCSF Chimera.^80^ Additional plots were made with seaborn.^81^

## SIGNIFICANCE

In this work we present MoltenProt, an open source software tool for robust analysis of high‐throughput protein thermal unfolding data. MoltenProt will be useful in diverse biochemical and structural biology applications, including buffer and construct optimization, functional analysis and small‐molecule screening.

## AUTHOR CONTRIBUTIONS

**Vadim Kotov:** Conceptualization; data curation; formal analysis; investigation; methodology; project administration; software; validation; visualization; writing‐original draft; writing‐review and editing. **Georg Mlynek:** Formal analysis; investigation; writing‐review and editing. **Oliver Vesper:** Investigation; writing‐review and editing. **Marina Pletzer:** Investigation; writing‐review and editing. **Jiri Wald:** Investigation; writing‐review and editing. **Celso M. Teixeira‐Duarte:** Investigation; resources; writing‐review and editing. **Herve Celia:** Investigation; resources; writing‐review and editing. **Maria Garcia‐Alai:** Investigation; methodology; resources; writing‐review and editing. **Stephan Nussberger:** Resources; writing‐review and editing. **Susan K. Buchanan:** Resources; writing‐review and editing. **João H. Morais‐Cabral:** Resources; writing‐review and editing. **Christian Loew:** Investigation; methodology; resources; writing‐review and editing. **Kristina Djinovic‐Carugo:** Methodology; resources; writing‐review and editing. **Thomas C. Marlovits:** Conceptualization; formal analysis; funding acquisition; methodology; writing‐original draft; writing‐review and editing.

## CONFLICT OF INTEREST

The authors declare no competing financial interests.

## Supporting information


**Supplementary Figure 1** MoltenProt software
Supplementary Figure 2: thermodynamic parameters obtained with MoltenProt agree well with literature data and orthogonal assays and raw data for Figure 2
Supplementary Figure 3: Interaction of intrinsically‐disordered protein FATZ‐1 with rod domain of α‐actinin‐2 and raw data for Figure 3
Supplementary Table 1: Comparison of widely used protein stabilization techniques
Supplementary Table 2: Comparison of thermodynamic parameters of lysozyme and RNAse A determined in this work with literature values
Supplementary Table 3: Comparison of thermodynamic characteristics of FlnC d19 obtained with CD, DSC and NanoDSF
Supplementary Table 4: Assessment of experimental errors in ΔH_m_, T_m_ and ΔG_u_°'
Supplementary Table 5: ΔH_m_, T_m_ and ΔG_u_ °′ values for samples presented in this work

Supplementary code.
