# Supplementary material for: In‐depth interrogation of protein thermal unfolding data with MoltenProt
Source: Protein Sci. 2020 Nov 21;30(1):201–17. doi: 10.1002/pro.3986 (PMC7737771; doi:10.1002/pro.3986)
Supplement: Supplementary file 1 — Supplementary Figure 1 MoltenProt software Supplementary Figure 2: thermodynamic parameters obtained with MoltenProt agree well with literature data and orthogonal assays and raw data for Figure 2 Supplementary Figure 3: Interaction of intrinsically‐disordered protein FATZ‐1 with rod domain of α‐actinin‐2 and raw data for Figure 3 Supplementary Table 1: Comparison of widely used protein stabilization techniques Supplementary Table 2: Comparison of thermodynamic parameters of lysozyme and RNAse A determined in this work with literature values Supplementary Table 3: Comparison of thermodynamic characteristics of FlnC d19 obtained with CD, DSC and NanoDSF Supplementary Table 4: Assessment of experimental errors in ΔHm, Tm and ΔGu°' Supplementary Table 5: ΔHm, Tm and ΔGu °′ values for samples presented in this work [file PRO-30-201-s001.pdf]

Supplementary figures

Supplementary figure 1: MoltenProt software

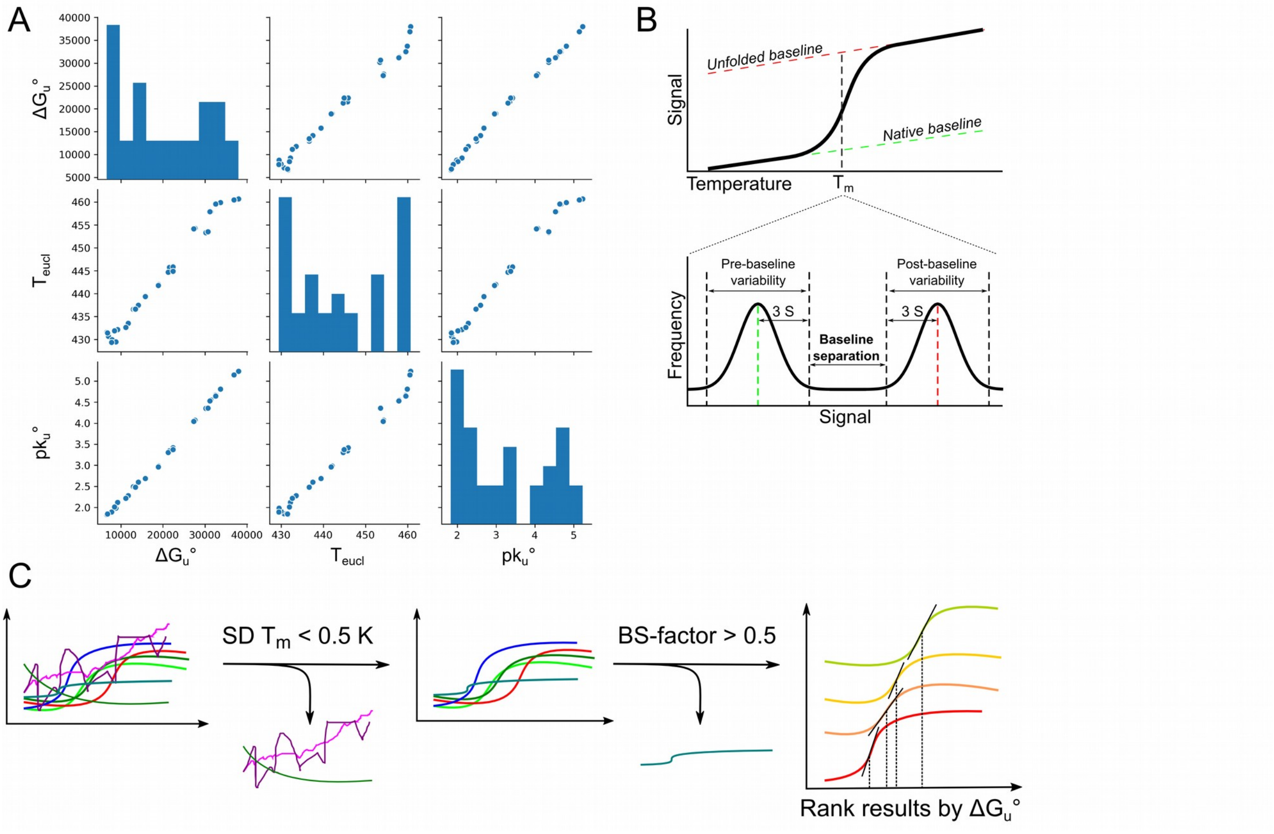

a) A pair-plot of ΔG_u°, pk_u° and T_{eucl} calculated from thermal unfolding curves of MdfA in diverse detergents. Non-diagonal plots show scatter-plots of respective two values, diagonal plots show a histograms of individual values.

b) The rationale for baseline separation factor (BS-factor). Baseline separation (BS) factor is a dimensionless measure to assess the height of an unfolding transition. At a given level of noise (approximated by standard error of estimate S) the pre- and post-transition baselines (green and red dashed lines) must be separated from each other by at least 6*S to make the unfolding transition distinguishable from noise. The higher the baseline separation the more reliably the transition can be fit. BS-factor is measured at T_m because at this temperature the folded and unfolded state are at equal concentrations.

c) Result filtering procedure implemented in MoltenProt. After the experimental curves are fit with the chosen equation the standard deviation (SD) of all fit parameters is calculated from the covariance matrix. Most invalid curves can be identified by high uncertainty of fit

of T_m. In addition, curves with suboptimal unfolding transitions are removed using a cutoff for BS-factor (see Materials and Methods).

Supplementary figure 2: thermodynamic parameters obtained with MoltenProt agree well with literature data and orthogonal assays and raw data for Figure 2

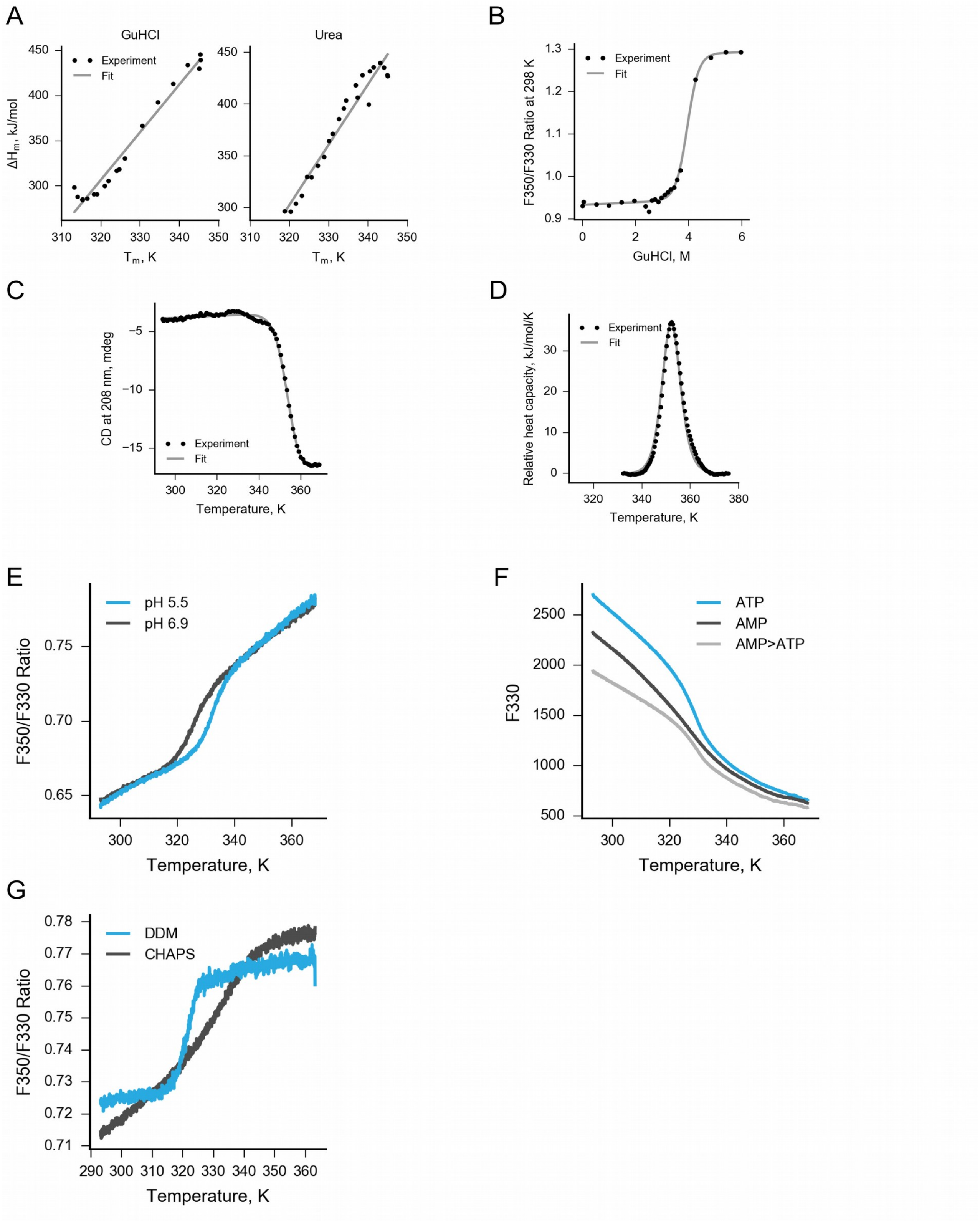

a) Determination of lysozyme ΔC_p by linear fitting of ΔH_m as a function of T_m. Dots indicate experimental data; lines indicate the linear fit.

b) Non-linear curve-fitting of lysozyme chemical unfolding data. Dots indicate experimental data; line indicates the fit.

c) Representative raw data (n=4) and fit of CD data for FlnC-d19.

d) Raw data and fit of DSC data for FlnC-d19; for the purposes of visualization, only every 15th datapoint is plotted.

e) Representative raw data (n=2) for thermal unfolding of ExbBD complex at pH 5.5 (blue) or pH 6.9 (gray).

f) Representative raw data for thermal unfolding of KtrAB complex in presence of ATP (blue, n=14) or AMP (gray, n=15). Light gray curve (AMP>ATP) corresponds to KtrAB purified in presence of AMP and subsequently incubated with 10 µM ATP to promote ligand exchange.

g) Representative raw data (n=2) for thermal unfolding of DgoT protein solubilized in DDM (blue) or CHAPS (gray)

Supplementary figure 3: Interaction of intrinsically-disordered protein FATZ-1 with rod domain of α-actinin-2 and raw data for Figure 3

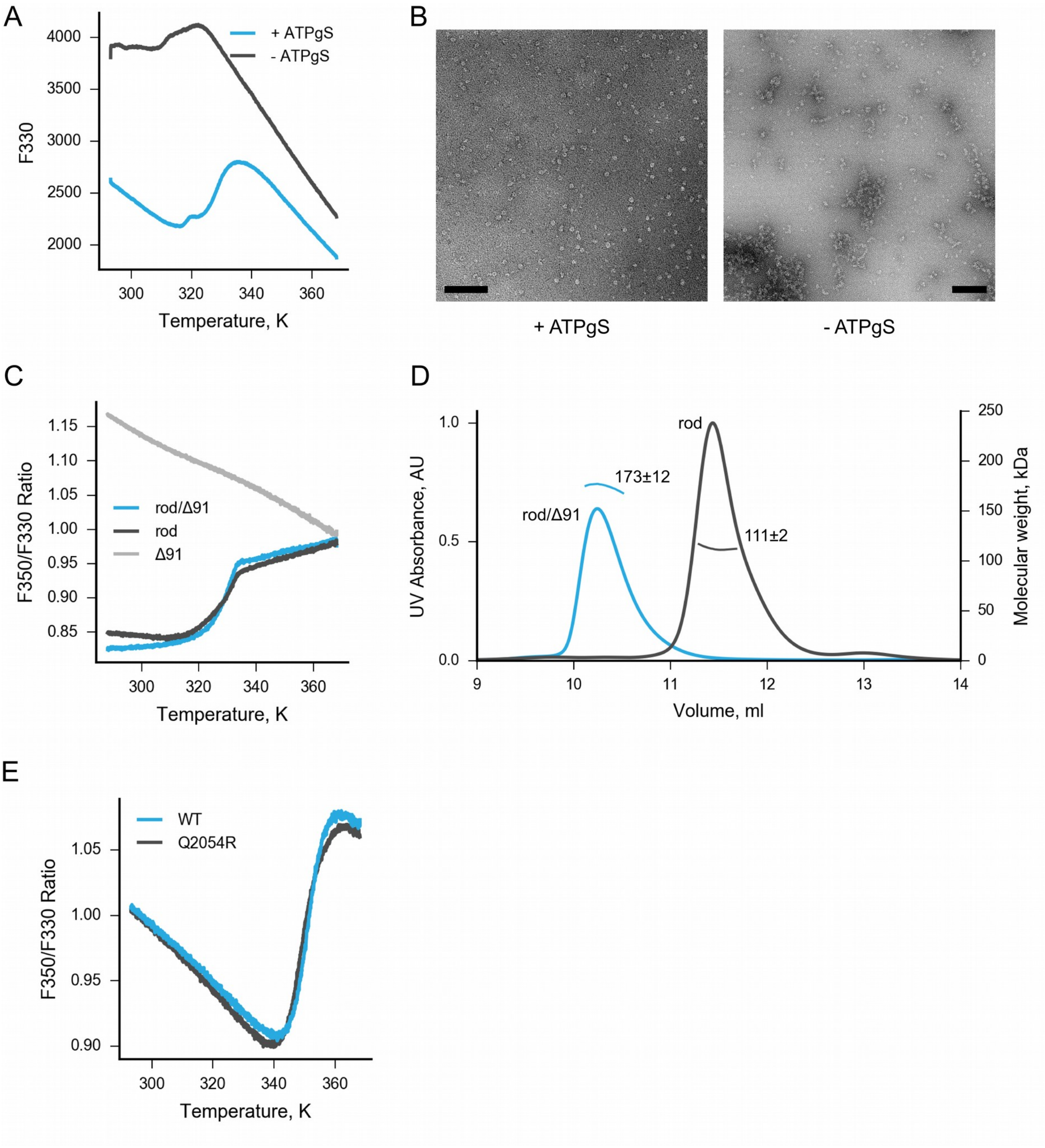

a) Representative raw data (n=2) for thermal unfolding of RuvB in presence (blue) or absence (gray) of 1 mM ATPgS.

b) full-sized micrographs of RuvB (scale bar 100 nm)

c) Representative raw data (n=3) for thermal unfolding of α-actinin-2 rod domain (gray), intrinsically disordered protein Δ91-FATZ-1 (light gray) and a complex thereof (blue).

d) SEC-MALLS analysis of eluates shows that a dimer of α-actinin-2 rod domain binds two Δ91-FATZ-1 molecules.

e) Representative raw data (n=3) for thermal unfolding of wild type of FlnC-d19 (blue) and cardiomyopathy causing mutation Q2058R (gray).

4 Supplementary tables

Supplementary Table 1: Comparison of widely used protein stabilization techniques

Assay name	Reference	Sample consumption	Samples per run	Min. sample volume, ul	Concentration range, mg/ml	Label-free	Buffer requirements	Sample requirements	Comment
CD	1	High	1	130	0.1-1	Yes	Low UV absorbance		CD plate readers are now available, but temperature control is poor
DSC	2	High	1	325	0.1-1	Yes	Unstable buffer components affect the baseline stability		
SEC and FSEC	3	Medium	1	10-30	0.1-1	Yes	Low absorbance and fluorescence at relevant wavelength	Presence of a fluorescent moiety for FSEC (e.g. GFP)	No thermodynamic information, high running costs
DSLS	4	Medium to low	96-1536	7-20	0.1-1	Yes	No compounds producing scattering signal (e.g. some detergents)	Aggregation must occur and be detectable	No thermodynamic information;<br>Signal depends on protein concentration
Thermofluor/ ProteoPlex	5	Medium	96-384	5-20	0.15-5	No	No detergents, EDTA	No heme-bearing proteins, no GFP-fusions	High uncertainty in ΔH_m values
CPM dye assay	6	Medium	96-384	5-20	0.15-5	No	No reducing agents (DTT, TCEP), fluorescence quenched by certain salts, decreased dye selectivity at pH above 8.0	Buried free cysteine residues in protein, no exposed free cysteins	Specificity of signal is under debate
Trp fluorescence (NanoDSF)	7	Medium to low	48	10-15	0.005-250	Yes	Low UV absorbance and fluorescence	At least one fluorescent amino acid (Trp of Tyr) that can report unfolding must be present in a relevant domain	Assay concentration depends on Trp content of the sample (protein concentration listed for IgG standard)

1 - Kelly SM, Jess TJ, Price NC. 2005. How to study proteins by circular dichroism. Biochimica et Biophysica Acta (BBA) - Proteins and Proteomics 1751:119–139.

2 - Johnson CM. 2013. Differential scanning calorimetry as a tool for protein folding and stability. Archives of Biochemistry and Biophysics, Protein Folding and Stability 531:100–109.

3 - Drew D, Lerch M, Kunji E, Slotboom D-J, de Gier J-W. 2006. Optimization of membrane protein overexpression and purification using GFP fusions. Nat Methods 3:303–313.

4 - Senisterra GA, Ghanei H, Khutoreskaya G, Dobrovetsky E, Edwards AM, Privé GG, Vedadi M. 2010. Assessing the stability of membrane proteins to detect ligand binding using differential static light scattering. J Biomol Screen 15:314–320.

5 - Chari A, Haselbach D, Kirves J-M, Ohmer J, Paknia E, Fischer N, Ganichkin O, Möller V, Frye JJ, Petzold G, Jarvis M, Tietzel M, Grimm C, Peters J-M, Schulman BA, Tittmann K, Markl J, Fischer U, Stark H. 2015. ProteoPlex: stability optimization of macromolecular complexes by sparse-matrix screening of chemical space. Nat Meth 12:859–865.

6 - Alexandrov AI, Mileni M, Chien EYT, Hanson MA, Stevens RC. 2008. Microscale Fluorescent Thermal Stability Assay for Membrane Proteins. Structure 16:351–359.

7 - Alexander CG, Wanner R, Johnson CM, Breitsprecher D, Winter G, Duhr S, Baaske P, Ferguson N. 2014. Novel microscale approaches for easy, rapid determination of protein stability in academic and commercial settings. Biochim Biophys Acta 1844:2241–2250.

Supplementary Table 2: Comparison of thermodynamic parameters of lysozyme and RNAse A determined in this work with literature values

		**Lysozyme**		**RNAse A**	
	Denaturation	*This work*	*Literature**	*This work*	*Literature**
ΔH_m, kJ/mol	Thermal	437.4±9.5	496-614	560.8±41.1	300-700
ΔC_p, J/mol/K	GuHCl	6709±1777.6	6276-6850	10353.1±1449.4	5000-8500
	Urea	5883.7±94.2		9852.1±2398.2	
$\Delta G_u^{\circ\prime}$, kJ/mol	GuHCl	42±5.1	37.18-73.6	-	32-80
	Thermal	59.9±1.5		63.5±4.6	
	Thermal, ΔCp corrected**	37.1±1.3		40.3±4.6	

$\Delta G_u^{\circ\prime}$ was not corrected for ΔC_p (see main text). Where applicable, values are given as average±standard deviation from n=2 replicate measurements of the same sample.
* the literature values vary, so a range is given. Literature values correspond to measurements at pH that is within +/- 1.5 pH unit of the buffer used in this work.
** ΔCp from GuHCl data was used.

Supplementary Table 3: Comparison of thermodynamic characteristics of FlnC d19 obtained with CD, DSC and NanoDSF

	**CD**	**DSC**	**NanoDSF**	**Predicted***
T_m, K	353.5±0.6	352.2±0.2	351±0.1	-
ΔC_p, J/mol/K	-	4910±823.4	4846.3±168.4	5742
ΔH_m, kJ/mol	363.7±34.9	401.5±3.4	350.6±2.3	-
ΔH_{cal}, kJ/mol	-	400.3±4.9	-	-
n	4	4	2	-

Where applicable, values are given as average±standard deviation from n replicate measurements of the same sample.

* FlnC-d19 contains 99 residues; ΔC_p formula from: Robertson AD, Murphy KP. 1997. Protein Structure and the Energetics of Protein Stability. Chem Rev 97:1251–1268.

Supplementary Table 4: Assessment of experimental errors in ΔH_m, T_m and $\Delta G_u^{\circ\prime}$

Sample	n	Sample concentration, mg/ml	Readout	ΔH_m, kJ/mol	T_m, K	$\Delta G_u^{\circ\prime}$, kJ/mol
KtrAB-ATP, day 1	5	0.1	F330	226.5±15.2	329.9±0.1	21.9±1.4
KtrAB-ATP, day 2	4	0.1	F330	212.1±7.8	329.8±0.2	20.4±0.9
KtrAB-ATP, day 3	5	0.1	F330	218.7±7.4	330.1±0.1	21.2±0.7
			All 3 days	219.1±7.2	329.9±0.2	21.2±0.7
			**Relative error, %**	**3.29%**	**0.05%**	**3.49%**
ExbBD, day 1	5	0.1	Ratio	383.5±56.8	319.2±0.6	25.4±3.3
ExbBD, day 2	5	0.1	Ratio	362±64.9	319.9±0.2	24.7±4.3
ExbBD, day 3	5	0.1	Ratio	399.3±43.4	318.5±0.4	25.6±2.4
			All 3 days	381.6±18.7	319.2±0.7	25.3±0.5
			**Relative error, %**	**4.90%**	**0.22%**	**1.82%**

Where applicable, values are given as average±standard deviation from n replicate measurements of the same sample.

Supplementary Table 5: ΔH_m, T_m and ΔG_u°′ values for samples presented in this work

Sample Name	Concentration, mg/ml	Buffer	Readout	n	ΔH_m, kJ/mol	T_m, K	ΔG_u°′, kJ/mol
ExbBD-pH 5.5	0.2	20 mM HEPES, pH 7.0, 150 mM NaCl, 0.01% NaN3, 0.08% C10E5 + MES pH 5.5 to 100 mM final	Ratio	2	302.9±69.5	332.9±1.16	31.7±6.3
ExbBD-pH 6.9	0.2	20 mM HEPES, pH 7.0, 150 mM NaCl, 0.01% NaN3, 0.08% C10E5 + MES pH 6.9 to 100 mM final	Ratio	2	286.5±11.1	324.9±0.61	23.7±0.4
KtrAB-ATP	0.1	20 mM Tris-HCl, pH 8.0, 120 mM NaCl, 30 mM KCl, 5 mM DTT, 10 uM ATP, 1.5 mM CYMAL-6	F330	14	219.1±7.2	329.9±0.15	21.2±0.7
KtrAB-AMP	0.1	20 mM Tris-HCl, pH 8.0, 120 mM NaCl, 30 mM KCl, 5 mM DTT, 1.5 mM CYMAL-6	F330	15	103.9±6.6	333.7±1.33	11.1±1
DgoT-DDM	0.5	20 mM HEPES, pH 7.5, 150 mM NaCl, 5% glycerol, 0.5 mM TCEP, 0.03% DDM	Ratio	2	471±1.7	321.3±0.04	34.1±0.2
DgoT-CHAPS	0.5	20 mM HEPES, pH 7.5, 150 mM NaCl, 5% glycerol, 0.5 mM TCEP, 1.2% CHAPS	Ratio	2	136.3±0.2	337.1±0.31	15.8±0.1
RuvB-ATPgS	0.5	20 mM Tris, pH 8.0, 20 mM NaCl, 10 mM MgCl2, 2% glycerol, 0.5 mM DTT, 1 mM ATPgS	F330	2	223.5±4.3	328.4±0.14	20.7±0.5
RuvB	0.5	20 mM Tris, pH 8.0, 20 mM NaCl, 10 mM MgCl2, 2% glycerol, 0.5 mM DTT	F330	2	242.2±3.5	318.3±0.04	15.5±0.2
Δ91-FATZ-1	0.6	20 mM Tris-HCl, 100 mM NaCl, 50 mM arginine, 50 mM glutamic acid, 0.5 mM PMSF, 1 mM EDTA, 1 mM β-mercaptoethanol, pH 7.5	Ratio	2	-	-	-
α-actinin-2 rod domain (rod)	0.48		Ratio	3	214.4±6.2	327.3±0.56	19.2±0.2
Rod/Δ91-FATZ-1 complex	0.38		Ratio	3	357.9±4.5	328.7±0.27	33.4±0.2
FLNc domain19 WT	0.83	9.6 mM Na/K phosphate buffer, pH 7.0, 137 mM NaCl, 2.7 mM KCl	Ratio	3	350.6±2.3	351±0.11	53±0.4
FLNc domain19 Q2058R	1.1	9.6 mM Na/K phosphate buffer, pH 7.0, 137 mM NaCl, 2.7 mM KCl	Ratio	3	334.5±1.8	348.9±0.06	48.8±0.3
TOM-core	0.25	20 mM HEPES, pH 7.2, 2% DMSO, 350 mM KCl, 0.1% DDM	Ratio	2	290.4±2.2	322.6±0.35	22.1±0.5
MdfA	0.5	20 mM HEPES, pH 7.5, 150 mM NaCl, 5% glycerol, 0.5 mM TCEP, 0.03% DDM	Ratio	2	341.5±5	331.6±0.11	34.6±0.6
Chicken egg lysozyme	0.1	50 mM sodium phosphate buffer, pH 7.5, 150 mM NaCl	Ratio	3	437.4±9.5	345.2±0.22	59.9±1.5
RNAse A from bovine pancreas	0.5	50 mM sodium phosphate buffer, pH 7.5, 150 mM NaCl	Ratio	4	560.8±41.1	336±0.03	63.5±4.6
Ubiquitin	0.84	10 mM sodium/potassium phosphate buffer, 137 mM NaCl, 2.7 mM KCl	Ratio	2	n/a	365±0.5	n/a

Where applicable, values are given as average±standard deviation from n replicate measurements of the same sample.
