# Supplementary material for: In‐depth interrogation of protein thermal unfolding data with MoltenProt
Source: Protein Sci. 2020 Nov 21;30(1):201–17. doi: 10.1002/pro.3986 (PMC7737771; doi:10.1002/pro.3986)
Supplement: Supplementary file 2 — Supplementary code. [file PRO-30-201-s002.zip › moltenprot/doc/index.html]

**MoltenProt
Documentation**

Vadim
Kotov

**Table
of Contents**

1.
What is MoltenProt? 2

2.
Step-by-step instructions 2

3.
Graphical user interface 4

3.1. Main window 4

3.2. Toolbar 4

3.2.1.
Readout combobox 4

3.2.2.
Heatmap combobox 5

3.3. File Menu 5

3.3.1.
File | New 5

3.3.2.
File | Open 5

3.3.3.
File | Export 5

3.3.4.
File | Save as JSON 5

3.3.5.
File | Quit 5

3.4. Actions |
Analysis 5

3.4.1.
Basic settings 5

3.4.2.
Pre-processing 6

3.4.3.
Misc. options 6

3.5. Actions | Edit
layout 7

3.6. Actions | Select
/ Deselect 7

3.7. Actions |
Settings 7

3.7.1.
Import 7

3.7.2.
Export 8

3.7.3.
Misc 8

3.7.4.
Plots 8

4.
Command-line interface 9

5.
Input/Output 10

5.1. Supported
formats 10

5.1.1.
CSV 10

5.1.2.
Layout CSV 10

5.1.3.
XLSX 10

5.1.4.
JSON 11

5.2. Output files 11

5.2.1.
Curves 11

5.2.2.
Fit parameters 11

5.2.3.
Sample information 12

6.
Models 13

6.1. Overview 13

6.2. Extensive and
intensive readouts 13

6.3. Equilibrium
models 13

6.3.1.
santoro1988 13

6.3.2.
santoro1988i 14

6.4. Empirical
models 14

6.4.1.
santoro1988d 15

6.4.2.
santoro1988di 15

6.5. Kinetic
models 15

6.5.1.
irrev 15

6.5.2.
lumry\_eyring 16

7.
Credits & copyright 17

7.1. MoltenProt 17

7.2. Dependencies 17

7.2.1.
pandas 17

7.2.2.
numpy 17

7.2.3.
scipy 18

7.2.4.
matplotlib 18

7.2.5.
PyQt5 18

7.2.6.
openpyxl 19

7.2.7.
xlrd 19

7.2.8.
joblib 21

7.2.9.
Oxygen icons 21

8.
References 22

# 1. What is MoltenProt?

MoltenProt is a program to fit sigmoidal curves
obtained with label-free protein unfolding assays, such as NanoDSF or
circular dichroism measurements. In addition to widely-used melting
temperature (Tm) MoltenProt uses other curve characteristics to rank
the results in terms of their (thermo)stability. Furthermore,
MoltenProt offers a panel of protein unfolding models, including
equilibrium unfolding, irreversible unfolding and the Lumry-Eyring
model. See Models for more
information.

MoltenProt provides a GUI
for exploratory data analysis and a CLI
for batch-processing.

# 2. Step-by-step instructions

1. Obtain a dataset, where protein unfolding is
   monitored with a label-free technique as a function of
   temperature.  
   **NOTE**: Two demo datasets (in CSV and XLSX
   format) are distributed together with MoltenProt.

   - If the input data is in XLSX format, use a
     spreadsheet editor to annotate the samples (sheet "Overview").

     - Start the MoltenProt GUI and load the
       dataset. Hover on individual samples with mouse to view the raw
       curves. Click on wells to display several curves. If multiple
       datasets are present in the file, a combobox will appear in the
       toolbar. Inspect the curves. If needed, set bad curve annotation to
       "Ignore" using Layout editor.

       - Open the analysis menu and select the model
         for each dataset. The default settings usually provide the best
         performance of the fit, however, the model may not reflect the real
         nature of protein unfolding.

         1. If curves contain spikes, they can be
            removed by trimming some values in the beginning or end of the
            curve. In more difficult cases, a median filter can be applied to
            smooth out the spikes.

            - If curves are too noisy, the signal strength
              may be improved by averaging datapoints to a larger degree step
              (shrinking).- Once the analysis is done, the sample
           stability will be color-coded on a heatmap. By default the
           model-supplied ranking parameter will be used for the heatmap. Other
           useful parameters for heatmap coloring will be available in a
           combobox. Click on the samples of interest to compare their fit
           curves side-by-side and show the fit parameters in a table.

           - Inspect highest/lowest ranked curves. How
             noisy is the measurement Does the fit result reflect the curve
             features?

             1. If a particularly noisy curve distorts the
                heatmap, it can be removed from analysis by annotating the sample
                as "Ignore" in Layout editor and re-running analysis.

                - If needed, perform fine-tuning of curve
                  fitting parameters.

                  - To store all analysis and visualization
                    settings save a MoltenProt session in JSON format. The session file
                    can be loaded later for re-analysis or data exporting.- Export the data using the format that is most
               appropriate for downstream analysis.

# 3. Graphical user interface

## 3.1. Main window

1. **Heatmap panel:** samples are color-coded
   with the selected curve characteristics. Hover-on with a mouse to
   visualize the data in the Plot window. Click several samples to
   visualize them side-by-side on the Plot window; their fit parameters
   will be shown in the Result table.

   - **Result table**: displays characteristics
     of selected curves. The set of characteristics to be displayed
     depends on the type of analysis performed.

     - **Plot window**: visualizes data requested
       by the user. A variety of plots can be displayed; use Settings
       for fine tuning.

       - **Protocol window**: displays the log of
         the data analysis including informational messages and warnings.
         Errors in analysis produce a pop-up window.

         - **Toolbar** and **menus** provide
           access to the functions of MoltenProt; **window decorator**
           is managed by the operating system.

## 3.2. Toolbar

### 3.2.1. Readout combobox

Switches between the readouts present in the input
file, e.g. F330, F350 and Ratio. If the input file contains a single
readout (e.g. plain CSV), then Readout combobox will not be shown.

### 3.2.2. Heatmap combobox

Selects a curve characteristic to color the
heatmap in the GUI. Available options depend on the chosen analysis
model.

### 3.2.3. Font settings

Loads the menu to adjust font size, type, etc in
MoltenProt. Useful for scaling the program window on high-resolution
displays.

## 3.3. File Menu

### 3.3.1. File | New

Start a new MoltenProt session.

### 3.3.2. File | Open

Open one of the supported
file formats: comma-separated values (CSV), NanoDSF processed
data (XLSX) or MoltenProt session (JSON).

### 3.3.3. File | Export

Export results with selected settings to a
directory.

### 3.3.4. File | Save as JSON

Save the current MoltenProt session.

### 3.3.5. File | Quit

Terminate the program.

## 3.4. Actions | Analysis

Set analysis settings and process data. OK button
will run the analysis, Cancel button will close the window, Reset to
defaults button will supply default values to all analysis
parameters.

### 3.4.1. Basic settings

This tab displays a table with available datasets
(1 in case of CSV, up to 5 in case of XLSX input file type) and a
combobox with possible models of analysis:

1. **santoro1988**: fast and robust fitting
   based on equilibrium two-state unfolding model;

   - **santoro1988i**: same as 1, but with an
     additional unfolding intermediate (three-state model);

     - **santoro1988d**: fast, but less robust
       fitting, which works descriptively, i.e. not assuming any unfolding
       mechanism; the idea is to provide the best quantitative description
       of the experimental curve;

       - **santoro1988di**: same as 3, but suitable
         for fitting unfolding curves with one intermediate (two peaks
         observed in the derivative plot);

         - **irrev**: irreversible two-state
           unfolding; protein unfolding is described kinetically, rather than
           using thermodynamics. This is a very common case, because most
           proteins do not reach equilibrium and unfold irreversibly.
           Computation requires numeric integration, so data processing is
           slow;

           - **lumry\_eyring**: Lumry-Eyring model for
             protein unfolding coupled with aggregation; can be used only if
             Scattering is available in the input data. First, the kinetics of
             aggregation are estimated using irrev model, and then unfolding and
             refolding kinetics are estimated. Computation requires numeric
             integration, so data processing is slow;

             - **skip**: the dataset will not be
               processed at all. The raw data will be preserved and can be
               re-analysed later on.

For more details on the models implemented in
MoltenProt see Models. In most cases
the choice of the model is the only decision required from the user.

### 3.4.2. Pre-processing

- **Median filtering**:
  remove spikes from the data by applying a median filter. The window
  size, i.e. the number of datapoints used to compute the median, is
  specified in temperature degrees. Median filter removes information
  from the data, and curve fitting is usually robust and not affected
  by spikes, so this option is rarely needed.

  - **Shrink data**:
    shrink data to a specified degree step. This step removes
    information from the data and decreases certainty of the fit, but
    may help expose global trends in the data. Also, shrunk data are
    processed faster.

    - **Remove from curve start/end**:
      drop some datapoints in the beginning of the end of the curve. This
      option may be helpful if the signal spikes
      at the start or end of the
      experiment.

### 3.4.3. Misc. options

- **Savitzky-Golay window size**:
  window size for Savitzky-Golay filter to calculate the smoothened
  first derivative. The window size is specified in temperature
  degrees and converted to an odd number of datapoints automatically.
  The smoothened derivative is used in data visualization
  and also provides the initial value for Tm.

  - **Data length for baseline estimation**:
    how many degrees in the beginning and the end of the curve are pure
    baselines, i.e. temperature dependence of the signal with 0% and
    100% protein molecules unfolded. The stretches of the data will be
    used to generate initial values for baseline fit parameters.

    - **Baseline bounds (n\*stdev)**:
      after initial baseline
      estimation (see previous option), MoltenProt uses the standard
      deviations for the slope and the intercept as the parameter bounds
      for pre- and post-transition baselines in the fitting of the full
      unfolding curve. In
      problematic cases this prevents the baselines from moving too far
      away from the experimental data.

      - **Heat capacity change (****Δ****C****p****)**:
        provide the value in J/mol/K, which will be used to extrapolate ΔGu
        from the unfolding region (around Tm)
        to the standard temperature
        of 298.15 K. If ΔCp
        is zero, then the extrapolation will be linear. For soluble proteins
        of 30 kDa and below ΔCp
        can be estimated by multiplying the number of residues by 58 [1].
        This value is only relevant for models based on equilibrium
        unfolding.

## 3.5. Actions | Edit layout

Create or edit annotations of individual samples.
This information will be shown in the GUI and, where applicable,
written to the output files. Annotations can be added manually via
the opened layout dialog or loaded from a CSV file. For XLSX files
the recommended way to supply annotations is to edit the "Overvew"
sheet in the original XLSX file with a spreadsheet editor. See
Supported Formats for
more info. Context menu for the layout dialog (right mouse button)
provides additional options:

- **Blank**:
  mark selected samples as blank buffer. During the analysis these
  curves will be averaged and subtracted from all other curves. This
  can be used to remove signal of the buffer. Note that the proper
  blank subtraction for F330/F350 Ratio data is not implemented.

  - **Ignore**:
    skip selected samples in the analysis.

    - **Clear selected cells**:
      clear any annotations in the selected cells.

## 3.6. Actions | Select / Deselect

Display/hide all curves in the dataset.

## 3.7. Actions | Settings

### 3.7.1. Import

- **CSV**:
  parameters for parsing unfolding data in CSV format:

  - Separator,
    Decimal separator: characters that separate data entries and
    indicate the decimal digit

    - Denaturation:
      indicate if the temperature scale in the input file is in Kelvins
      or Celsius or that the data is chemical denaturation.

      - Scan rate:
        heating rate in degrees/min. Scan rate is relevant only for
        non-equilibrium models.- **XLSX**:
    parameters for parsing XLSX data (NanoDSF):

    - Refolding data:
      Indicate if the refolding ramp was used in the experiment; this is
      needed for correct parsing of input files, however, the refolding
      data itself is not used in downstream analysis.

### 3.7.2. Export

- **Data table format**:

  - "\*.csv":
    output comma-separated files with UTF encoding and each table will
    be an individual file.

    - "\*.xlsx":
      export a single file with multiple sheets.- **Figures/heatmaps**:
    include images of individual plots or heatmaps in the output. The
    colormap for the heatmap will
    be same as in the GUI.

    - **Report type**:

      - "None":
        no reports are generated.

        - "Compact
          XLSX": produce an XLSX file containing a result table for all
          readouts, but no curves or images will be generated.

          - "Interactive
            HTML": the report will contain a full package of data
            including XLSX files with raw, fit, baseline-corrected
            curves, analysis results
            and standard deviations. The files will be linked in a single HTML
            file, which also allows viewing plots of individual samples. The
            main advantage of HTML reports is that they do not require
            installation of MoltenProt and can be viewed in any modern
            web-browser.

For more details see Output
files.

### 3.7.3. Misc

- **Parallel processes**:
  MoltenProt will can some steps with the specified number of
  subprocesses. This can speed up the workflow, but will also consume
  more computer resources.

  - **Colormap for heatmap**:
    Choose one of the matplotlib colormaps for the main heatmap:  
      
    Colormaps with "\_r" will have inverse color
    direction.

### 3.7.4. Plots

- **Display curve**:

  - "Experimental
    signal": show raw experimental data.

    - "Baseline-corrected":
      pre- and post-transition baselines are subtracted from the data to
      get a sigmoidal curve ranging from 0 to 1. This viewing mode
      simplifies comparison between samples.- **Display as**:

    - "Datapoints
      + Fit": show both experimental and the fit result.

      - "Datapoints":
        only experimental data.

        - "Fit":
          only fit data.- **Baselines**: show pre- and
      post-transition baselines obtained in the fitting as dashed lines.
      If one of the baselines is not visible or is too far from the data,
      then the fitting may have gone wrong.

      - **Vertical lines**:
        show characteristic
        temperatures (e.g. Tm
        or T\_onset)
        as vertical lines on the
        plot.

        - **Show every**:
          Show only n-th experimental datapoint. This is useful to unclutter
          dense experimental curves.

          - **Derivative plot**:
            Create an additional plot showing the smoothened first derivative of
            experimental data.

            - **Legend**:
              Show legend for the plot.

# 4. Command-line interface

Upon installation, MoltenProt is accessible as a
Python module:

```
python -m moltenprot --help
```

All options implemented in the GUI are also available via the CLI.
The only difference is that per-readout model settings are not
available. The main usage for CLI is to perform processing of
multiple datasets with the same parameters:

```
python -m moltenprot -i dataset1.xlsx dataset2.xlsx -o all_datasets -rj 2 --exclude_readout 330nm --model_sct santoro1988d
```

This command will process files dataset1.xlsx and dataset2.xlsx,
write the output (HTML report) to folder all\_datasets. The readout
called "330nm" will be excluded and for scattering data
model santoro1988d will be applied. All other datasets will be
processed with default model santoro1988. The program will run in two
parallel processes.

# 5. Input/Output

## 5.1. Supported formats

### 5.1.1. CSV

Comma-separated value (CSV) file for MoltenProt
must follow several rules:

- First row contains column names.

  - One column is called "Temperature"
    and contains the X-axis values.

    - All other columns have an alphanumeric index
      similar to a 96-well plate (from A1 to H12).

```
Temperature,A1,A2, ... ,H12
20,1300,1500,...,1600
21,1400,1600,...,1700
...
95,2000,2100,...,2200
```

Under the hood MoltenProt uses pandas.read\_csv to parse CSV files, so
any separator supported by this module can be used in MoltenProt.

### 5.1.2. Layout CSV

These files provide a description of individual
samples and can be added to MoltenProt session with Layout
editor. Files should follow several requirements:

- First row contains column names.

  - One column is called "ID" and
    contains an alphanumeric index similar to a 96-well plate (from A1
    to H12).

    - One column is called "Condition"
      and contains the annotations.

      - The file can be only comma-separated, any
        other separators are not allowed; it is also recommended that all
        text is quoted.

        - An optional column can be called "dCp"
          and can contain per-sample values of heat capacity change.

```
"ID","Condition"
"A1","Ultrapure water"
"A2","Original buffer"
...
"H12","Blank"
```

### 5.1.3. XLSX

XLSX files with "processed" data
generated by PR.ThermControl (NanoTemper GmbH, tested with v.2.1.2)
can be opened directly in MoltenProt. The annotations provided in the
"Overview" sheet are imported as well. While MoltenProt
offers basic capabilities for editing annotations, it is recommended
to set all annotations in the "Overview" sheet using a
full-featured spreadsheet editor.

**NOTE:** An
additional readout, F350-F330 (deltaF), is computed automatically.
For more info see
Models section.

### 5.1.4. JSON

MoltenProt uses JavaScript Object Notation (JSON)
format to store sessions, i.e. the current state of the program.
Sessions contain raw and processed data as well as annotations,
analysis options used, timestamps etc, thus providing an easy way to
save results for later viewing.

## 5.2. Output files

File export settings can be adjusted in
Settings|Export. MoltenProt
usually writes out multiple files, so it is recommended to export
results to a dedicated directory. The exception is saving of JSON
sessions, where everything is written into a single file.

### 5.2.1. Curves

For each readout present in the input file (e.g.
F330, F350, Ratio) CSV export will produce a separate file for each
curve type (see below). XLSX export will create a single XLSX file
for each readout present in the input file. The following curve types
are available:

- **Raw data**
  (not exported to CSV): original data read from the input file.

  - **Preprocessed data**
    (CSV files with suffix "\_preproc\_curves"): raw data that
    underwent the pre-processing procedure, such as median filtering or
    shrinking (see Analysis).
    The curves will have blanks subtracted (if any specified in the
    layout) and samples marked as "Ignore" in the layout will
    be removed.

    - **Fit curves**
      (CSV files with suffix "\_fit"): these curves are computed
      over the whole X-axis range of the input data using the determined
      fit parameters and are used to generate plots in the GUI and PNG
      format. This table is provided for convenience in case plotting of
      fit data outside MoltenProt is planned.

      - **Baseline-corrected**
        (CSV files with suffix "\_raw\_corr"):
        raw data corrected for the pre- and post-transition baselines
        determined by the fit.
        These curves are
        useful for visualization and comparison between samples, because all
        Y-axis values are always
        in range from 0 (no protein unfolded) to 1 (all protein unfolded).

### 5.2.2. Fit parameters

The results of the fit are presented in two
separate XLSX sheets/CSV files:

- **F****it parameters** (CSV files with
  suffix "\_results"): a table with all curve characteristics
  computed by MoltenProt. Parameters with suffix "\_init" are
  the initial parameters for the non-linear curve-fitting procedure;
  suffix "\_fit" marks the parameters obtained with curve
  fitting.

  - **Standard deviations** (CSV files with
    suffix "\_results\_stdev"): non-linear curve-fitting
    procedure also yields a covariance matrix for obtained fit
    parameters. This information can be used to estimate the uncertainty
    of the fit and thus conclude if the current fitting result is
    reliable. For instance, well fit curves have Tm standard deviation
    of 0.5 K or below.

Initial values of the fit parameters have suffix
"\_init", fit results have suffix "\_fit". Some
parameters are shared by all built-in models of MoltenProt:

- **kN, bN**: slope and intercept of the
  pre-transition baseline, i.e. temperature dependence of native state
  (N) fluorescence.

  - **kU, bU**: slope and intercept of the
    post-transition baseline, i.e. temperature dependence of unfolded
    state (U) fluorescence.

    - **S**: standard error of the estimate (in
      units of Y-axis). This metric assesses how well the experimental
      data is described by the fit: for 99% of datapoints the difference
      between the observed and fit values will be below 3\*S. S is very
      sensitive for large outliers, such as spikes in the signal, so it
      should not be used as a single value to assess the curve quality

      - **BS\_factor**: baseline separation factor
        (unit-less). BS-factor is a quantitative measure to assess how far
        is the pre-transition baseline from the post-transition baseline at
        Tm taking into account the noise in the signal (estimated via S).
        Curves with BS-factor above 0.8 are exceptionally good, while curves
        with BS-factor in range 0-0.5 should be treated with caution.
        Negative BS-factor means that the curve is not suitable for
        interpretation. Since BS-factor is relative and unit-less, it is
        particularly helpful in deciding which readout to use for downstream
        analysis (e.g. F330 vs Ratio in NanoDSF data): the readout with
        higher average BS-factor is more preferable.

Model-specific parameters are described in Models
section.

### 5.2.3. Sample information

The following sample information is included in
the result table:

- **ID**: internal sample number assigned by
  MoltenProt. Up to 96 samples can be processed at once (A1 to H12).

  - **Capillary**
    (only NanoDSF data):
    capillary position in the device.

    - **Condition**: annotation describing the
      contents of the sample. For NanoDSF data the value is read from
      sheet "Overview". Annotations can be changed via Layout
      editor.

# 6. Models

## 6.1. Overview

MoltenProt implements a variety of protein
unfolding models, which should cover most common use-cases. All
models rely on linear baseline extrapolation, which also means that
data with sloping baselines should be processed with caution. This
section contains a brief theoretical background for each model and
introduces the recommended measure for final result ranking. All
ranking measures are chosen in such a way that higher values
correspond to higher stability of the protein.

## 6.2. Extensive and intensive readouts

Strictly
speaking, the models implemented in MoltenProt are only applicable to
extensive readouts, i.e. when the signal is proportional
to protein concentration.
While this is the case for raw fluorescence (330 or 350 nm) and
Scattering, the F350/F330 Ratio is an intensive readout, because it
is a proxy
for
the shape of the fluorescence spectrum. Applying
equations for an extensive readout to an intensive readout produces
an additional systematic
error [2], [3]. The Ratio readout, however, tends to produce the most
clean and easy to interpret
sigmoidal curves, so
omitting it from analysis decreases the explanatory power of the
assay. For NanoDSF data MoltenProt also calculates deltaF readout
(difference between fluorescence at 350 and 330 nm), which represents
a trade-off between correctness and robustness. On the one hand,
deltaF is an extensive readout, because it is a linear combination of
F330 and F350. On the other hand, subtraction of fluorescences
removes a significant part of the baseline drift and can make the
unfolding transition more pronounced.

## 6.3. Equilibrium models

Equilibrium models rely on several assumptions: 1)
protein unfolding is a reversible reaction; 2) at every timepoint of
the measurement the system is at chemical equilibrium; 3) protein
heat capacity change (ΔCp)
is temperature-independent.

### 6.3.1. santoro1988

This model (applied to chemical denaturation) was
initially described in ref. [4]. A more elaborate discussion for
derivation of formulas is in ref. [3]. The model assumes that the
protein exists in either native (N) or unfolded (U) state and there
is an equilibrium between the folding and unfolding reactions (N
⇆ U).
The law of signal F(T) is described by equation:

where kN, bN are slope and intercept of the
pre-transition (native) baseline, kU bU are slope and intercept of
post-transition baseline, R is the universal gas constant, ΔHm
is enthalpy of unfolding at melting temperature Tm.

The final
ranking metric is dG\_std:
Gibbs free energy of unfolding extrapolated to the standard state
temperature (298.15 K). dG\_std integrates the slope and the
inflection point of an unfolding curve into a single measure.

By default
extrapolation to the standard state temperature is linear, which is
equal to the assumption that ΔCp
is zero. If ΔCp
is known, the
extrapolated dG\_std can be corrected by adding ΔCp
\* dCp\_component.
dCp\_component is automatically calculated in santoro1988 mode and
added to the output.

### 6.3.2. santoro1988i

This model is based on the same assumptions as the
previous one, however, three states are possible for the protein:
native (N), unfolded (U) and unfolding intermediate (I). If the Tm
for N and I states is significantly different, it is possible to see
two unfolding transitions in the experimental curve (two peaks on the
smoothened derivative curve). Derivation is also described in ref
[5]. The law of signal is as follows:

where kN, bN are slope and intercept of the
pre-transition (native) baseline, kU bU are slope and intercept of
post-transition baseline, kI is the signal slope for the I state (the
state is assumed to be short-lived, so the intercept is not
modelled), R is the universal gas constant, ΔHm1
and ΔHm2
are enthalpy of unfolding
at melting temperature T1
and T2 (melting temperature for N ⇆
I and I ⇆
U reactions).

The final
ranking metric is dG\_comb\_std:
Gibbs free energy of unfolding extrapolated to the standard state
temperature (298.15 K), which is a sum of dG\_std for reactions N
⇆ I
and I ⇆ U
(thermodynamic coupling).

## 6.4. Empirical models

Empirical models describe sigmoidal curves that
are common in thermal unfolding assays without providing insights
about the properties of the protein molecules.

### 6.4.1. santoro1988d

This model is a "descriptive" version of
santoro1988 model: instead of enthalpy of unfolding at Tm (ΔHm),
the model uses onset temperature T\_onset to describe the steepness of
the curve. T\_onset is the temperature at which 1% of protein is
unfolded. The exponent in santoro1988 model is thus substituted to
the following expression:

The final ranking metric is the square root of the
sum of squared Tm and T\_onset. This can be thought of as the
Euclidean distance from the point 0,0 K of a scatter plot between Tm
and T\_onset. The samples that are most far away from this point will
have the most beneficial combination of Tm and T\_onset. This ranking
assumes that Tm and T\_onset are equally important for protein
stability.

See also ref. [6].

### 6.4.2. santoro1988di

This model is a "descriptive" version of
santoro1988i model. The exponents with ΔHm1-2
and Tm1-2 are substituted to exponents using T\_onset1-2 and Tm1-2
(see santoro1988d). Similarly to santoro1988i,
this model can describe unfolding curves with two transitions.

The
final ranking metric is the sum of geometric means of Tm1 and
T\_onset1
and Tm2 and T\_onset2.

## 6.5. Kinetic models

Kinetic models describe protein unfolding
reactions via reaction rate constant, which links conversion of
reactants to products with time. Arrhenius equation is used to model
the temperature dependence of the reaction rate constant.

### 6.5.1. irrev

This model assumes that protein exists in states N
and U only and the unfolding reaction is irreversible (N → U). The
law of signal F(T) is defined as follows:

where kN, bN are slope and intercept of the
pre-transition (native) baseline, kU bU are slope and intercept of
post-transition baseline and xN(T) is fraction of natively folded
molecules as a function of temperature. xN(T) is obtained via numeric
integration:

where Tmin and Tmax are the start and end
temperatures of the measurement, v is the scan rate (degrees/min), Ea
is activation energy of unfolding, Tf is the temperature where
reaction rate constant of unfolding (k) equals 1, R is the universal
gas constant. xN is assumed to be 1 at Tmin. See refs [5], [7] for
derivation of similar equations for differential scanning calorimetry
data.

The final ranking metric for this model is the
negative logarithm of the reaction rate constant at standard state
temperature (pk\_std). Similarly to dG\_std in santoro1988, this metric
integrates slope and inflection point of the unfolding curve
(represented here as Ea and Tf) in a single measure of stability.

### 6.5.2. lumry\_eyring

The Lumry-Eyring model [8] assumes that the
protein exists in three states: native (N), unfolded (U) and
aggregated (A). The reaction U → A is irreversible. Two more
non-equilibrium reactions N → U and U → N with reaction rate
constants kF and kR describe the transition from state N to state U.
Fitting the whole model to typical experimental data is not stable,
so in MoltenProt this model is applied in two steps:

1. Scattering data (part of NanoDSF datasets if
   the respective detector is available) is fit with irrev model to
   obtain activation energy Ea and Tf (temperature where the rate
   constant is 1) for reaction U → A. It is assumed that states and N
   and U produce the same Scattering signal.

   - The obtained parameters are supplied to the
     equation that describes protein unfolding signal F(T) (e.g.
     F350/F330 ratio in NanoDSF data) as a function of fraction native
     state xN, fraction unfolded state xU and fraction aggregated state
     xA.

where kN, bN are slope and intercept of the
pre-transition (native) baseline, kU bU are slope and intercept of
the post-transition baseline (which is in fact represented by the A
state, but not U), kI is the slope for the U state of unfolding. The
equations for xU and xA are obtained with numeric integration [5].

The final ranking metric for this model is the
negative logarithm of the ratio of the reaction rate constants for
reactions N → U and U → N calculated at standard state
temperature (pk\_ratio\_std). Similarly to dG\_std in santoro1988, this
metric integrates slope and inflection point of the unfolding curve
in a single measure of stability. The special feature of lumry\_eyring
model in MoltenProt is that it can integrate not only the information
from individual readouts, but also combine the stability as measured
by Scattering with one selected unfolding readout.

# 7. Credits & copyright

## 7.1. MoltenProt

```
Copyright 2018,2019,2020 Vadim Kotov, Thomas C. Marlovits
    MoltenProt is free software: you can redistribute it and/or modify
    it under the terms of the GNU General Public License as published by
    the Free Software Foundation, either version 3 of the License, or
    (at your option) any later version.

    MoltenProt is distributed in the hope that it will be useful,
    but WITHOUT ANY WARRANTY; without even the implied warranty of
    MERCHANTABILITY or FITNESS FOR A PARTICULAR PURPOSE.  See the
    GNU General Public License for more details.

    You should have received a copy of the GNU General Public License
    along with MoltenProt.  If not, see <https://www.gnu.org/licenses/>.
```

## 7.2. Dependencies

### 7.2.1. pandas

https://pandas.pydata.org/

```
Copyright (c) 2008-2011, AQR Capital Management, LLC, Lambda Foundry, Inc. and PyData Development Team
All rights reserved.

Copyright (c) 2011-2020, Open source contributors.

Redistribution and use in source and binary forms, with or without
modification, are permitted provided that the following conditions are met:

* Redistributions of source code must retain the above copyright notice, this
  list of conditions and the following disclaimer.

* Redistributions in binary form must reproduce the above copyright notice,
  this list of conditions and the following disclaimer in the documentation
  and/or other materials provided with the distribution.

* Neither the name of the copyright holder nor the names of its
  contributors may be used to endorse or promote products derived from
  this software without specific prior written permission.

THIS SOFTWARE IS PROVIDED BY THE COPYRIGHT HOLDERS AND CONTRIBUTORS "AS IS"
AND ANY EXPRESS OR IMPLIED WARRANTIES, INCLUDING, BUT NOT LIMITED TO, THE
IMPLIED WARRANTIES OF MERCHANTABILITY AND FITNESS FOR A PARTICULAR PURPOSE ARE
DISCLAIMED. IN NO EVENT SHALL THE COPYRIGHT HOLDER OR CONTRIBUTORS BE LIABLE
FOR ANY DIRECT, INDIRECT, INCIDENTAL, SPECIAL, EXEMPLARY, OR CONSEQUENTIAL
DAMAGES (INCLUDING, BUT NOT LIMITED TO, PROCUREMENT OF SUBSTITUTE GOODS OR
SERVICES; LOSS OF USE, DATA, OR PROFITS; OR BUSINESS INTERRUPTION) HOWEVER
CAUSED AND ON ANY THEORY OF LIABILITY, WHETHER IN CONTRACT, STRICT LIABILITY,
OR TORT (INCLUDING NEGLIGENCE OR OTHERWISE) ARISING IN ANY WAY OUT OF THE USE
OF THIS SOFTWARE, EVEN IF ADVISED OF THE POSSIBILITY OF SUCH DAMAGE.
```

### 7.2.2. numpy

http://numpy.org/

```
Copyright © 2005-2020, NumPy Developers.
All rights reserved.
Redistribution and use in source and binary forms, with or without modification, are permitted provided that the following conditions are met:

* Redistributions of source code must retain the above copyright notice, this
  list of conditions and the following disclaimer.

* Redistributions in binary form must reproduce the above copyright notice,
  this list of conditions and the following disclaimer in the documentation
  and/or other materials provided with the distribution.

* Neither the name of the copyright holder nor the names of its
  contributors may be used to endorse or promote products derived from
  this software without specific prior written permission.

THIS SOFTWARE IS PROVIDED BY THE COPYRIGHT HOLDERS AND CONTRIBUTORS "AS IS"
AND ANY EXPRESS OR IMPLIED WARRANTIES, INCLUDING, BUT NOT LIMITED TO, THE
IMPLIED WARRANTIES OF MERCHANTABILITY AND FITNESS FOR A PARTICULAR PURPOSE ARE
DISCLAIMED. IN NO EVENT SHALL THE COPYRIGHT HOLDER OR CONTRIBUTORS BE LIABLE
FOR ANY DIRECT, INDIRECT, INCIDENTAL, SPECIAL, EXEMPLARY, OR CONSEQUENTIAL
DAMAGES (INCLUDING, BUT NOT LIMITED TO, PROCUREMENT OF SUBSTITUTE GOODS OR
SERVICES; LOSS OF USE, DATA, OR PROFITS; OR BUSINESS INTERRUPTION) HOWEVER
CAUSED AND ON ANY THEORY OF LIABILITY, WHETHER IN CONTRACT, STRICT LIABILITY,
OR TORT (INCLUDING NEGLIGENCE OR OTHERWISE) ARISING IN ANY WAY OUT OF THE USE
OF THIS SOFTWARE, EVEN IF ADVISED OF THE POSSIBILITY OF SUCH DAMAGE.
```

### 7.2.3. scipy

https://scipy.org/

```
Copyright © 2001, 2002 Enthought, Inc.
All rights reserved.

Copyright © 2003-2019 SciPy Developers.
All rights reserved.
Redistribution and use in source and binary forms, with or without modification, are permitted provided that the following conditions are met:
```

- ```
  Redistributions of source code must retain the above copyright notice, this list of conditions and the following disclaimer.
  ```

  - ```
    Redistributions in binary form must reproduce the above copyright notice, this list of conditions and the following disclaimer in the documentation and/or other materials provided with the distribution.
    ```

    - ```
      Neither the name of Enthought nor the names of the SciPy Developers may be used to endorse or promote products derived from this software without specific prior written permission.
      ```

```
THIS SOFTWARE IS PROVIDED BY THE COPYRIGHT HOLDERS AND CONTRIBUTORS "AS IS" AND ANY EXPRESS OR IMPLIED WARRANTIES, INCLUDING, BUT NOT LIMITED TO, THE IMPLIED WARRANTIES OF MERCHANTABILITY AND FITNESS FOR A PARTICULAR PURPOSE ARE DISCLAIMED. IN NO EVENT SHALL THE REGENTS OR CONTRIBUTORS BE LIABLE FOR ANY DIRECT, INDIRECT, INCIDENTAL, SPECIAL, EXEMPLARY, OR CONSEQUENTIAL DAMAGES (INCLUDING, BUT NOT LIMITED TO, PROCUREMENT OF SUBSTITUTE GOODS OR SERVICES; LOSS OF USE, DATA, OR PROFITS; OR BUSINESS INTERRUPTION) HOWEVER CAUSED AND ON ANY THEORY OF LIABILITY, WHETHER IN CONTRACT, STRICT LIABILITY, OR TORT (INCLUDING NEGLIGENCE OR OTHERWISE) ARISING IN ANY WAY OUT OF THE USE OF THIS SOFTWARE, EVEN IF ADVISED OF THE POSSIBILITY OF SUCH DAMAGE.
```

### 7.2.4. matplotlib

https://matplotlib.org/

"Copyright (c) 2012-2013 Matplotlib
Development Team; All Rights Reserved"

### 7.2.5. PyQt5

https://www.riverbankcomputing.com/software/pyqt/

```
PyQt5 is copyright (c) Riverbank Computing Limited.
THERE IS NO WARRANTY FOR THE PROGRAM, TO THE EXTENT PERMITTED BY
APPLICABLE LAW.  EXCEPT WHEN OTHERWISE STATED IN WRITING THE COPYRIGHT
HOLDERS AND/OR OTHER PARTIES PROVIDE THE PROGRAM "AS IS" WITHOUT WARRANTY
OF ANY KIND, EITHER EXPRESSED OR IMPLIED, INCLUDING, BUT NOT LIMITED TO,
THE IMPLIED WARRANTIES OF MERCHANTABILITY AND FITNESS FOR A PARTICULAR
PURPOSE.  THE ENTIRE RISK AS TO THE QUALITY AND PERFORMANCE OF THE PROGRAM
IS WITH YOU.  SHOULD THE PROGRAM PROVE DEFECTIVE, YOU ASSUME THE COST OF
ALL NECESSARY SERVICING, REPAIR OR CORRECTION.
```

### 7.2.6. openpyxl

https://openpyxl.readthedocs.io/en/stable/

```
Copyright (c) 2010 openpyxl

Permission is hereby granted, free of charge, to any person obtaining a
copy of this software and associated documentation files (the
"Software"), to deal in the Software without restriction, including
without limitation the rights to use, copy, modify, merge, publish,
distribute, sublicense, and/or sell copies of the Software, and to
permit persons to whom the Software is furnished to do so, subject to
the following conditions:

The above copyright notice and this permission notice shall be included
in all copies or substantial portions of the Software.

THE SOFTWARE IS PROVIDED "AS IS", WITHOUT WARRANTY OF ANY KIND, EXPRESS
OR IMPLIED, INCLUDING BUT NOT LIMITED TO THE WARRANTIES OF
MERCHANTABILITY, FITNESS FOR A PARTICULAR PURPOSE AND NONINFRINGEMENT.
IN NO EVENT SHALL THE AUTHORS OR COPYRIGHT HOLDERS BE LIABLE FOR ANY
CLAIM, DAMAGES OR OTHER LIABILITY, WHETHER IN AN ACTION OF CONTRACT,
TORT OR OTHERWISE, ARISING FROM, OUT OF OR IN CONNECTION WITH THE
SOFTWARE OR THE USE OR OTHER DEALINGS IN THE SOFTWARE.

Odict implementation in openpyxl/writer/odict.py uses the following licence:

Copyright (c) 2001-2011 Python Software Foundation
              2011 Raymond Hettinger
License: PYTHON SOFTWARE FOUNDATION LICENSE VERSION 2
         See http://www.opensource.org/licenses/Python-2.0 for full terms
Note: backport changes by Raymond were originally distributed under MIT
      license, but since the original license for Python is more 
      restrictive than MIT, code cannot be released under its terms and
      still adheres to the limitations of Python license.
```

### 7.2.7. xlrd

https://pypi.org/project/xlrd/

```
There are two licenses associated with xlrd. This one relates to the bulk of
the work done on the library::

    Portions copyright © 2005-2009, Stephen John Machin, Lingfo Pty Ltd
    All rights reserved.

    Redistribution and use in source and binary forms, with or without
    modification, are permitted provided that the following conditions are met:

    1. Redistributions of source code must retain the above copyright notice,
    this list of conditions and the following disclaimer.

    2. Redistributions in binary form must reproduce the above copyright notice,
    this list of conditions and the following disclaimer in the documentation
    and/or other materials provided with the distribution.

    3. None of the names of Stephen John Machin, Lingfo Pty Ltd and any
    contributors may be used to endorse or promote products derived from this
    software without specific prior written permission.

    THIS SOFTWARE IS PROVIDED BY THE COPYRIGHT HOLDERS AND CONTRIBUTORS "AS IS"
    AND ANY EXPRESS OR IMPLIED WARRANTIES, INCLUDING, BUT NOT LIMITED TO,
    THE IMPLIED WARRANTIES OF MERCHANTABILITY AND FITNESS FOR A PARTICULAR
    PURPOSE ARE DISCLAIMED. IN NO EVENT SHALL THE COPYRIGHT OWNER OR CONTRIBUTORS
    BE LIABLE FOR ANY DIRECT, INDIRECT, INCIDENTAL, SPECIAL, EXEMPLARY, OR
    CONSEQUENTIAL DAMAGES (INCLUDING, BUT NOT LIMITED TO, PROCUREMENT OF
    SUBSTITUTE GOODS OR SERVICES; LOSS OF USE, DATA, OR PROFITS; OR BUSINESS
    INTERRUPTION) HOWEVER CAUSED AND ON ANY THEORY OF LIABILITY, WHETHER IN
    CONTRACT, STRICT LIABILITY, OR TORT (INCLUDING NEGLIGENCE OR OTHERWISE)
    ARISING IN ANY WAY OUT OF THE USE OF THIS SOFTWARE, EVEN IF ADVISED OF
    THE POSSIBILITY OF SUCH DAMAGE.

This one covers some earlier work::

    /*-
     * Copyright (c) 2001 David Giffin.
     * All rights reserved.
     *
     * Based on the the Java version: Andrew Khan Copyright (c) 2000.
     *
     *
     * Redistribution and use in source and binary forms, with or without
     * modification, are permitted provided that the following conditions
     * are met:
     *
     * 1. Redistributions of source code must retain the above copyright
     *    notice, this list of conditions and the following disclaimer.
     *
     * 2. Redistributions in binary form must reproduce the above copyright
     *    notice, this list of conditions and the following disclaimer in
     *    the documentation and/or other materials provided with the
     *    distribution.
     *
     * 3. All advertising materials mentioning features or use of this
     *    software must display the following acknowledgment:
     *    "This product includes software developed by
     *     David Giffin <david@giffin.org>."
     *
     * 4. Redistributions of any form whatsoever must retain the following
     *    acknowledgment:
     *    "This product includes software developed by
     *     David Giffin <david@giffin.org>."
     *
     * THIS SOFTWARE IS PROVIDED BY DAVID GIFFIN ``AS IS'' AND ANY
     * EXPRESSED OR IMPLIED WARRANTIES, INCLUDING, BUT NOT LIMITED TO, THE
     * IMPLIED WARRANTIES OF MERCHANTABILITY AND FITNESS FOR A PARTICULAR
     * PURPOSE ARE DISCLAIMED.  IN NO EVENT SHALL DAVID GIFFIN OR
     * ITS CONTRIBUTORS BE LIABLE FOR ANY DIRECT, INDIRECT, INCIDENTAL,
     * SPECIAL, EXEMPLARY, OR CONSEQUENTIAL DAMAGES (INCLUDING, BUT
     * NOT LIMITED TO, PROCUREMENT OF SUBSTITUTE GOODS OR SERVICES;
     * LOSS OF USE, DATA, OR PROFITS; OR BUSINESS INTERRUPTION)
     * HOWEVER CAUSED AND ON ANY THEORY OF LIABILITY, WHETHER IN CONTRACT,
     * STRICT LIABILITY, OR TORT (INCLUDING NEGLIGENCE OR OTHERWISE)
     * ARISING IN ANY WAY OUT OF THE USE OF THIS SOFTWARE, EVEN IF ADVISED
     * OF THE POSSIBILITY OF SUCH DAMAGE.
     *
```

### 7.2.8. joblib

https://joblib.readthedocs.io/

```
Copyright (c) 2008-2016, The joblib developers.
All rights reserved.

Redistribution and use in source and binary forms, with or without
modification, are permitted provided that the following conditions are met:

* Redistributions of source code must retain the above copyright notice, this
  list of conditions and the following disclaimer.

* Redistributions in binary form must reproduce the above copyright notice,
  this list of conditions and the following disclaimer in the documentation
  and/or other materials provided with the distribution.

* Neither the name of the copyright holder nor the names of its
  contributors may be used to endorse or promote products derived from
  this software without specific prior written permission.

THIS SOFTWARE IS PROVIDED BY THE COPYRIGHT HOLDERS AND CONTRIBUTORS "AS IS"
AND ANY EXPRESS OR IMPLIED WARRANTIES, INCLUDING, BUT NOT LIMITED TO, THE
IMPLIED WARRANTIES OF MERCHANTABILITY AND FITNESS FOR A PARTICULAR PURPOSE ARE
DISCLAIMED. IN NO EVENT SHALL THE COPYRIGHT HOLDER OR CONTRIBUTORS BE LIABLE
FOR ANY DIRECT, INDIRECT, INCIDENTAL, SPECIAL, EXEMPLARY, OR CONSEQUENTIAL
DAMAGES (INCLUDING, BUT NOT LIMITED TO, PROCUREMENT OF SUBSTITUTE GOODS OR
SERVICES; LOSS OF USE, DATA, OR PROFITS; OR BUSINESS INTERRUPTION) HOWEVER
CAUSED AND ON ANY THEORY OF LIABILITY, WHETHER IN CONTRACT, STRICT LIABILITY,
OR TORT (INCLUDING NEGLIGENCE OR OTHERWISE) ARISING IN ANY WAY OUT OF THE USE
OF THIS SOFTWARE, EVEN IF ADVISED OF THE POSSIBILITY OF SUCH DAMAGE.
```

### 7.2.9. Oxygen icons

https://github.com/KDE/oxygen-icons

```
The Oxygen Icon Theme
    Copyright (C) 2007 Nuno Pinheiro <nuno@oxygen-icons.org>
    Copyright (C) 2007 David Vignoni <david@icon-king.com>
    Copyright (C) 2007 David Miller <miller@oxygen-icons.org>
    Copyright (C) 2007 Johann Ollivier Lapeyre <johann@oxygen-icons.org>
    Copyright (C) 2007 Kenneth Wimer <kwwii@bootsplash.org>
    Copyright (C) 2007 Riccardo Iaconelli <riccardo@oxygen-icons.org>
    

and others

    This library is free software; you can redistribute it and/or
    modify it under the terms of the GNU Lesser General Public
    License as published by the Free Software Foundation; either
    version 3 of the License, or (at your option) any later version.

    This library is distributed in the hope that it will be useful,
    but WITHOUT ANY WARRANTY; without even the implied warranty of
    MERCHANTABILITY or FITNESS FOR A PARTICULAR PURPOSE.  See the GNU
    Lesser General Public License for more details.

    You should have received a copy of the GNU Lesser General Public
    License along with this library. If not, see <http://www.gnu.org/licenses/>.

Clarification:

  The GNU Lesser General Public License or LGPL is written for
  software libraries in the first place. We expressly want the LGPL to
  be valid for this artwork library too.

  KDE Oxygen theme icons is a special kind of software library, it is an
  artwork library, it's elements can be used in a Graphical User Interface, or
  GUI.

  Source code, for this library means:
   - where they exist, SVG;
   - otherwise, if applicable, the multi-layered formats xcf or psd, or
  otherwise png.

  The LGPL in some sections obliges you to make the files carry
  notices. With images this is in some cases impossible or hardly useful.

  With this library a notice is placed at a prominent place in the directory
  containing the elements. You may follow this practice.

  The exception in section 5 of the GNU Lesser General Public License covers
  the use of elements of this art library in a GUI.

  kde-artists [at] kde.org
```

# 8. References

[1] A. D. Robertson and K. P. Murphy, “Protein Structure and the
Energetics of Protein Stability,” *Chem. Rev.*, vol. 97, no.
5, pp. 1251–1268, Aug. 1997, doi: 10.1021/cr960383c.

[2] E. Monsellier and H. Bedouelle, “Quantitative measurement of
protein stability from unfolding equilibria monitored with the
fluorescence maximum wavelength,” *Protein Eng Des Sel*, vol.
18, no. 9, pp. 445–456, Sep. 2005, doi: 10.1093/protein/gzi046.

[3] H. Bedouelle, “Principles and equations for measuring and
interpreting protein stability: From monomer to tetramer,”
*Biochimie*, vol. 121, pp. 29–37, Feb. 2016, doi:
10.1016/j.biochi.2015.11.013.

[4] M. M. Santoro and D. W. Bolen, “Unfolding free energy changes
determined by the linear extrapolation method. 1. Unfolding of
phenylmethanesulfonyl .alpha.-chymotrypsin using different
denaturants,” *Biochemistry*, vol. 27, no. 21, pp. 8063–8068,
Oct. 1988, doi: 10.1021/bi00421a014.

[5] S. Mazurenko, A. Kunka, K. Beerens, C. M. Johnson, J. Damborsky,
and Z. Prokop, “Exploration of Protein Unfolding by Modelling
Calorimetry Data from Reheating,” *Sci Rep*, vol. 7, Nov.
2017, doi: 10.1038/s41598-017-16360-y.

[6] V. Kotov *et al.*, “High-throughput stability screening
for detergent-solubilized membrane proteins,” *Scientific
Reports*, vol. 9, no. 1, p. 10379, Jul. 2019, doi:
10.1038/s41598-019-46686-8.

[7] J. M. Sanchez-Ruiz, “Theoretical analysis of Lumry-Eyring
models in differential scanning calorimetry,” *Biophys J*,
vol. 61, no. 4, pp. 921–935, Apr. 1992.

[8] R. Lumry and H. Eyring, “Conformation Changes of Proteins,”
*J. Phys. Chem.*, vol. 58, no. 2, pp. 110–120, Feb. 1954,
doi: 10.1021/j150512a005.
