# Supplementary material for: In‐depth interrogation of protein thermal unfolding data with MoltenProt
Source: Protein Sci. 2020 Nov 21;30(1):201–17. doi: 10.1002/pro.3986 (PMC7737771; doi:10.1002/pro.3986)
Supplement: Supplementary file 2 — Supplementary code. [file PRO-30-201-s002.zip › moltenprot/doc/index.pdf]

MoltenProt Documentation

Vadim Kotov

Table of Contents

1. What is MoltenProt?....2
2. Step-by-step instructions....2
3. Graphical user interface....4
   3.1. Main window....4
   3.2. Toolbar....4
      3.2.1. Readout combobox....4
      3.2.2. Heatmap combobox....5
   3.3. File Menu....5
      3.3.1. File | New....5
      3.3.2. File | Open....5
      3.3.3. File | Export....5
      3.3.4. File | Save as JSON....5
      3.3.5. File | Quit....5
   3.4. Actions | Analysis....5
      3.4.1. Basic settings....5
      3.4.2. Pre-processing....6
      3.4.3. Misc. options....6
   3.5. Actions | Edit layout....7
   3.6. Actions | Select / Deselect....7
   3.7. Actions | Settings....7
      3.7.1. Import....7
      3.7.2. Export....8
      3.7.3. Misc....8
      3.7.4. Plots....8
4. Command-line interface....9
5. Input/Output....10
   5.1. Supported formats....10
      5.1.1. CSV....10
      5.1.2. Layout CSV....10
      5.1.3. XLSX....10
      5.1.4. JSON....11
   5.2. Output files....11
      5.2.1. Curves....11
      5.2.2. Fit parameters....11
      5.2.3. Sample information....12
6. Models....13
   6.1. Overview....13
   6.2. Extensive and intensive readouts....13
   6.3. Equilibrium models....13
      6.3.1. santoro1988....13
      6.3.2. santoro1988i....14
   6.4. Empirical models....14
      6.4.1. santoro1988d....15

6.4.2. santoro1988di....15
6.5. Kinetic models....15
6.5.1. irrev....15
6.5.2. lumry_eyring....16
7. Credits & copyright....17
7.1. MoltenProt....17
7.2. Dependencies....17
7.2.1. pandas....17
7.2.2. numpy....17
7.2.3. scipy....18
7.2.4. matplotlib....18
7.2.5. PyQt5....18
7.2.6. openpyxl....19
7.2.7. xlrd....19
7.2.8. joblib....21
7.2.9. Oxygen icons....21
8. References....22

1. What is MoltenProt?

MoltenProt is a program to fit sigmoidal curves obtained with label-free protein unfolding assays, such as NanoDSF or circular dichroism measurements. In addition to widely-used melting temperature (Tm) MoltenProt uses other curve characteristics to rank the results in terms of their (thermo)stability. Furthermore, MoltenProt offers a panel of protein unfolding models, including equilibrium unfolding, irreversible unfolding and the Lumry-Eyring model. See Models for more information.

MoltenProt provides a GUI for exploratory data analysis and a CLI for batch-processing.

2. Step-by-step instructions

1. Obtain a dataset, where protein unfolding is monitored with a label-free technique as a function of temperature.
   NOTE: Two demo datasets (in CSV and XLSX format) are distributed together with MoltenProt.
2. If the input data is in XLSX format, use a spreadsheet editor to annotate the samples (sheet "Overview").
3. Start the MoltenProt GUI and load the dataset. Hover on individual samples with mouse to view the raw curves. Click on wells to display several curves. If multiple datasets are present in the file, a combobox will appear in the toolbar. Inspect the curves. If needed, set bad curve annotation to "Ignore" using Layout editor.

4. Open the analysis menu and select the model for each dataset. The default settings usually provide the best performance of the fit, however, the model may not reflect the real nature of protein unfolding.
    4.1. If curves contain spikes, they can be removed by trimming some values in the beginning or end of the curve. In more difficult cases, a median filter can be applied to smooth out the spikes.
    4.2. If curves are too noisy, the signal strength may be improved by averaging datapoints to a larger degree step (shrinking).
5. Once the analysis is done, the sample stability will be color-coded on a heatmap. By default the model-supplied ranking parameter will be used for the heatmap. Other useful parameters for heatmap coloring will be available in a combobox. Click on the samples of interest to compare their fit curves side-by-side and show the fit parameters in a table.
6. Inspect highest/lowest ranked curves. How noisy is the measurement Does the fit result reflect the curve features?
    6.1. If a particularly noisy curve distorts the heatmap, it can be removed from analysis by annotating the sample as "Ignore" in Layout editor and re-running analysis.
    6.2. If needed, perform fine-tuning of curve fitting parameters.
    6.3. To store all analysis and visualization settings save a MoltenProt session in JSON format. The session file can be loaded later for re-analysis or data exporting.
7. Export the data using the format that is most appropriate for downstream analysis.

3. Graphical user interface

3.1. Main window

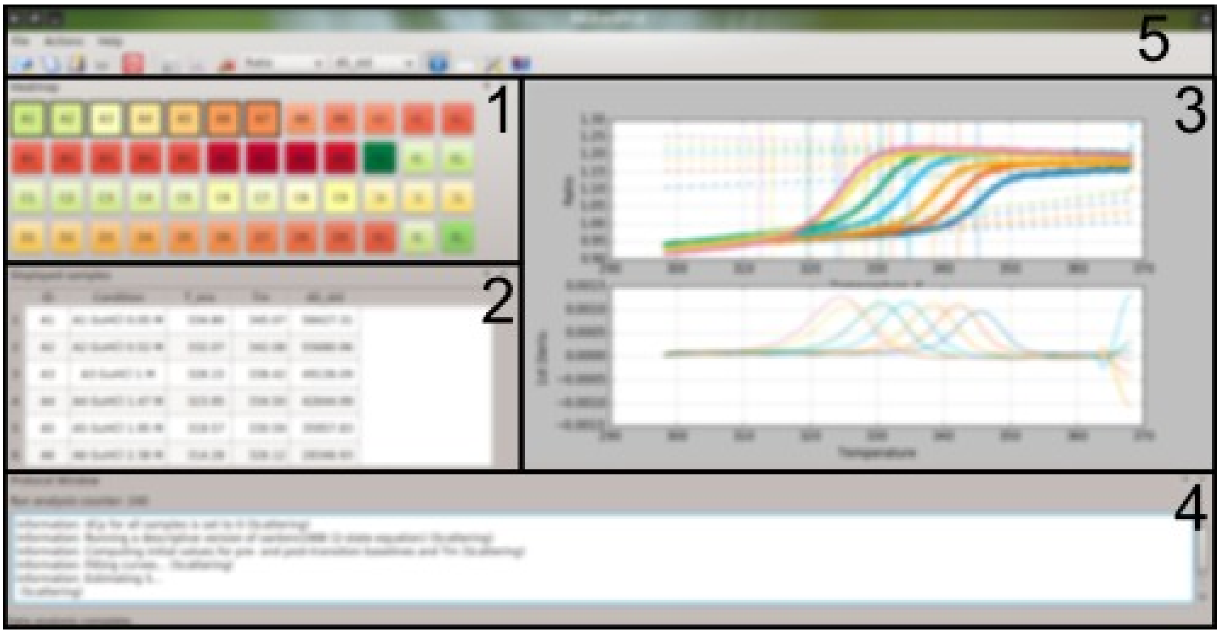

1. **Heatmap panel:** samples are color-coded with the selected curve characteristics. Hover-on with a mouse to visualize the data in the Plot window. Click several samples to visualize them side-by-side on the Plot window; their fit parameters will be shown in the Result table.
2. **Result table**: displays characteristics of selected curves. The set of characteristics to be displayed depends on the type of analysis performed.
3. **Plot window**: visualizes data requested by the user. A variety of plots can be displayed; use Settings for fine tuning.
4. **Protocol window**: displays the log of the data analysis including informational messages and warnings. Errors in analysis produce a pop-up window.
5. **Toolbar** and **menus** provide access to the functions of MoltenProt; **window decorator** is managed by the operating system.

3.2. Toolbar

3.2.1. Readout combobox

Switches between the readouts present in the input file, e.g. F330, F350 and Ratio. If the input file contains a single readout (e.g. plain CSV), then Readout combobox will not be shown.

3.2.2. Heatmap combobox

Selects a curve characteristic to color the heatmap in the GUI. Available options depend on the chosen analysis model.

3.2.3. Font settings

Loads the menu to adjust font size, type, etc in MoltenProt. Useful for scaling the program window on high-resolution displays.

3.3. File Menu

3.3.1. File | New

Start a new MoltenProt session.

3.3.2. File | Open

Open one of the supported file formats: comma-separated values (CSV), NanoDSF processed data (XLSX) or MoltenProt session (JSON).

3.3.3. File | Export

Export results with selected settings to a directory.

3.3.4. File | Save as JSON

Save the current MoltenProt session.

3.3.5. File | Quit

Terminate the program.

3.4. Actions | Analysis

Set analysis settings and process data. OK button will run the analysis, Cancel button will close the window, Reset to defaults button will supply default values to all analysis parameters.

3.4.1. Basic settings

This tab displays a table with available datasets (1 in case of CSV, up to 5 in case of XLSX input file type) and a combobox with possible models of analysis:

1. **santoro1988**: fast and robust fitting based on equilibrium two-state unfolding model;
2. **santoro1988i**: same as 1, but with an additional unfolding intermediate (three-state model);
3. **santoro1988d**: fast, but less robust fitting, which works descriptively, i.e. not assuming any unfolding mechanism; the idea is to provide the best quantitative description of the experimental curve;

4. **santoro1988di**: same as 3, but suitable for fitting unfolding curves with one intermediate (two peaks observed in the derivative plot);
5. **irrev**: irreversible two-state unfolding; protein unfolding is described kinetically, rather than using thermodynamics. This is a very common case, because most proteins do not reach equilibrium and unfold irreversibly. Computation requires numeric integration, so data processing is slow;
6. **lumry_eyring**: Lumry-Eyring model for protein unfolding coupled with aggregation; can be used only if Scattering is available in the input data. First, the kinetics of aggregation are estimated using irrev model, and then unfolding and refolding kinetics are estimated. Computation requires numeric integration, so data processing is slow;
7. **skip**: the dataset will not be processed at all. The raw data will be preserved and can be re-analysed later on.

For more details on the models implemented in MoltenProt see Models. In most cases the choice of the model is the only decision required from the user.

3.4.2. Pre-processing

- **Median filtering**: remove spikes from the data by applying a median filter. The window size, i.e. the number of datapoints used to compute the median, is specified in temperature degrees. Median filter removes information from the data, and curve fitting is usually robust and not affected by spikes, so this option is rarely needed.
- **Shrink data**: shrink data to a specified degree step. This step removes information from the data and decreases certainty of the fit, but may help expose global trends in the data. Also, shrunk data are processed faster.
- **Remove from curve start/end**: drop some datapoints in the beginning of the end of the curve. This option may be helpful if the signal spikes at the start or end of the experiment.

3.4.3. Misc. options

- **Savitzky-Golay window size**: window size for Savitzky-Golay filter to calculate the smoothened first derivative. The window size is specified in temperature degrees and converted to an odd number of datapoints automatically. The smoothened derivative is used in data visualization and also provides the initial value for Tm.
- **Data length for baseline estimation**: how many degrees in the beginning and the end of the curve are pure baselines, i.e. temperature dependence of the signal with 0% and 100% protein molecules unfolded.

The stretches of the data will be used to generate initial values for baseline fit parameters.

- **Baseline bounds (n*stdev)**: after initial baseline estimation (see previous option), MoltenProt uses the standard deviations for the slope and the intercept as the parameter bounds for pre- and post-transition baselines in the fitting of the full unfolding curve. In problematic cases this prevents the baselines from moving too far away from the experimental data.
- **Heat capacity change (ΔCp)**: provide the value in J/mol/K, which will be used to extrapolate ΔGu from the unfolding region (around Tm) to the standard temperature of 298.15 K. If ΔCp is zero, then the extrapolation will be linear. For soluble proteins of 30 kDa and below ΔCp can be estimated by multiplying the number of residues by 58 [1]. This value is only relevant for models based on equilibrium unfolding.

3.5. Actions | Edit layout

Create or edit annotations of individual samples. This information will be shown in the GUI and, where applicable, written to the output files. Annotations can be added manually via the opened layout dialog or loaded from a CSV file. For XLSX files the recommended way to supply annotations is to edit the "Overvew" sheet in the original XLSX file with a spreadsheet editor. See Supported Formats for more info. Context menu for the layout dialog (right mouse button) provides additional options:

- **Blank**: mark selected samples as blank buffer. During the analysis these curves will be averaged and subtracted from all other curves. This can be used to remove signal of the buffer. Note that the proper blank subtraction for F330/F350 Ratio data is not implemented.
- **Ignore**: skip selected samples in the analysis.
- **Clear selected cells**: clear any annotations in the selected cells.

3.6. Actions | Select / Deselect

Display/hide all curves in the dataset.

3.7. Actions | Settings

3.7.1. Import

- **CSV**: parameters for parsing unfolding data in CSV format:
  - Separator, Decimal separator: characters that separate data entries and indicate the decimal digit

    - Denaturation: indicate if the temperature scale in the input file is in Kelvins or Celsius or that the data is chemical denaturation.
    - Scan rate: heating rate in degrees/min. Scan rate is relevant only for non-equilibrium models.
- **XLSX**: parameters for parsing XLSX data (NanoDSF):
    - Refolding data: Indicate if the refolding ramp was used in the experiment; this is needed for correct parsing of input files, however, the refolding data itself is not used in downstream analysis.

3.7.2. Export

- **Data table format**:
    - "*.csv": output comma-separated files with UTF encoding and each table will be an individual file.
    - "*.xlsx": export a single file with multiple sheets.
- **Figures/heatmaps**: include images of individual plots or heatmaps in the output. The colormap for the heatmap will be same as in the GUI.
- **Report type**:
    - "None": no reports are generated.
    - "Compact XLSX": produce an XLSX file containing a result table for all readouts, but no curves or images will be generated.
    - "Interactive HTML": the report will contain a full package of data including XLSX files with raw, fit, baseline-corrected curves, analysis results and standard deviations. The files will be linked in a single HTML file, which also allows viewing plots of individual samples. The main advantage of HTML reports is that they do not require installation of MoltenProt and can be viewed in any modern web-browser.

For more details see Output files.

3.7.3. Misc

- **Parallel processes**: MoltenProt will can some steps with the specified number of subprocesses. This can speed up the workflow, but will also consume more computer resources.
- **Colormap for heatmap**: Choose one of the matplotlib colormaps for the main heatmap:

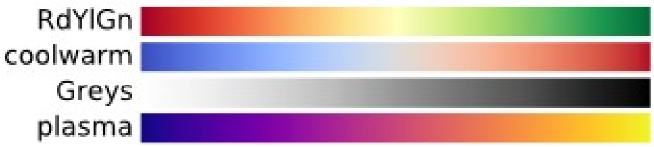

Colormaps with "_r" will have inverse color direction.

3.7.4. Plots

- **Display curve**:
  - "Experimental signal": show raw experimental data.
  - "Baseline-corrected": pre- and post-transition baselines are subtracted from the data to get a sigmoidal curve ranging from 0 to 1. This viewing mode simplifies comparison between samples.
- **Display as**:
  - "Datapoints + Fit": show both experimental and the fit result.
  - "Datapoints": only experimental data.
  - "Fit": only fit data.
- **Baselines**: show pre- and post-transition baselines obtained in the fitting as dashed lines. If one of the baselines is not visible or is too far from the data, then the fitting may have gone wrong.
- **Vertical lines**: show characteristic temperatures (e.g. Tm or T_onset) as vertical lines on the plot.
- **Show every**: Show only n-th experimental datapoint. This is useful to unclutter dense experimental curves.
- **Derivative plot**: Create an additional plot showing the smoothened first derivative of experimental data.
- **Legend**: Show legend for the plot.

4. Command-line interface

Upon installation, MoltenProt is accessible as a Python module:

```
python -m moltenprot --help
```

All options implemented in the GUI are also available via the CLI. The only difference is that per-readout model settings are not available. The main usage for CLI is to perform processing of multiple datasets with the same parameters:

```
python -m moltenprot -i dataset1.xlsx dataset2.xlsx -o all_datasets -rj 2
--exclude_readout 330nm --model_sct santoro1988d
```

This command will process files dataset1.xlsx and dataset2.xlsx, write the output (HTML report) to folder all_datasets. The readout called "330nm" will be

excluded and for scattering data model santoro1988d will be applied. All other datasets will be processed with default model santoro1988. The program will run in two parallel processes.

5. Input/Output

5.1. Supported formats

5.1.1. CSV

Comma-separated value (CSV) file for MoltenProt must follow several rules:

- First row contains column names.
- One column is called "Temperature" and contains the X-axis values.
- All other columns have an alphanumeric index similar to a 96-well plate (from A1 to H12).

```
Temperature,A1,A2, ... ,H12
20,1300,1500,...,1600
21,1400,1600,...,1700
...
95,2000,2100,...,2200
```

Under the hood MoltenProt uses pandas.read_csv to parse CSV files, so any separator supported by this module can be used in MoltenProt.

5.1.2. Layout CSV

These files provide a description of individual samples and can be added to MoltenProt session with Layout editor. Files should follow several requirements:

- First row contains column names.
- One column is called "ID" and contains an alphanumeric index similar to a 96-well plate (from A1 to H12).
- One column is called "Condition" and contains the annotations.
- The file can be only comma-separated, any other separators are not allowed; it is also recommended that all text is quoted.
- An optional column can be called "dCp" and can contain per-sample values of heat capacity change.

```
"ID","Condition"
"A1","Ultrapure water"
"A2","Original buffer"
...
"H12","Blank"
```

5.1.3. XLSX

XLSX files with "processed" data generated by PR.ThermControl (NanoTemper GmbH, tested with v.2.1.2) can be opened directly in MoltenProt. The annotations provided in the "Overview" sheet are imported as well. While MoltenProt offers basic capabilities for editing annotations, it is recommended to set all annotations in the "Overview" sheet using a full-featured spreadsheet editor.

NOTE: An additional readout, F350-F330 (deltaF), is computed automatically. For more info see Models section.

5.1.4. JSON

MoltenProt uses JavaScript Object Notation (JSON) format to store sessions, i.e. the current state of the program. Sessions contain raw and processed data as well as annotations, analysis options used, timestamps etc, thus providing an easy way to save results for later viewing.

5.2. Output files

File export settings can be adjusted in Settings|Export. MoltenProt usually writes out multiple files, so it is recommended to export results to a dedicated directory. The exception is saving of JSON sessions, where everything is written into a single file.

5.2.1. Curves

For each readout present in the input file (e.g. F330, F350, Ratio) CSV export will produce a separate file for each curve type (see below). XLSX export will create a single XLSX file for each readout present in the input file. The following curve types are available:

- **Raw data** (not exported to CSV): original data read from the input file.
- **Preprocessed data** (CSV files with suffix "_preproc_curves"): raw data that underwent the pre-processing procedure, such as median filtering or shrinking (see Analysis). The curves will have blanks subtracted (if any specified in the layout) and samples marked as "Ignore" in the layout will be removed.
- **Fit curves** (CSV files with suffix "_fit"): these curves are computed over the whole X-axis range of the input data using the determined fit parameters and are used to generate plots in the GUI and PNG format. This table is provided for convenience in case plotting of fit data outside MoltenProt is planned.
- **Baseline-corrected** (CSV files with suffix "_raw_corr"): raw data corrected for the pre- and post-transition baselines determined by the fit. These curves are useful for visualization and comparison between

samples, because all Y-axis values are always in range from 0 (no protein unfolded) to 1 (all protein unfolded).

5.2.2. Fit parameters

The results of the fit are presented in two separate XLSX sheets/CSV files:

- **Fit parameters** (CSV files with suffix "_results"): a table with all curve characteristics computed by MoltenProt. Parameters with suffix "_init" are the initial parameters for the non-linear curve-fitting procedure; suffix "_fit" marks the parameters obtained with curve fitting.
- **Standard deviations** (CSV files with suffix "_results_stdev"): non-linear curve-fitting procedure also yields a covariance matrix for obtained fit parameters. This information can be used to estimate the uncertainty of the fit and thus conclude if the current fitting result is reliable. For instance, well fit curves have Tm standard deviation of 0.5 K or below.

Initial values of the fit parameters have suffix "_init", fit results have suffix "_fit". Some parameters are shared by all built-in models of MoltenProt:

- **kN, bN**: slope and intercept of the pre-transition baseline, i.e. temperature dependence of native state (N) fluorescence.
- **kU, bU**: slope and intercept of the post-transition baseline, i.e. temperature dependence of unfolded state (U) fluorescence.
- **S**: standard error of the estimate (in units of Y-axis). This metric assesses how well the experimental data is described by the fit: for 99% of datapoints the difference between the observed and fit values will be below 3*S. S is very sensitive for large outliers, such as spikes in the signal, so it should not be used as a single value to assess the curve quality
- **BS_factor**: baseline separation factor (unit-less). BS-factor is a quantitative measure to assess how far is the pre-transition baseline from the post-transition baseline at Tm taking into account the noise in the signal (estimated via S). Curves with BS-factor above 0.8 are exceptionally good, while curves with BS-factor in range 0-0.5 should be treated with caution. Negative BS-factor means that the curve is not suitable for interpretation. Since BS-factor is relative and unit-less, it is particularly helpful in deciding which readout to use for downstream analysis (e.g. F330 vs Ratio in NanoDSF data): the readout with higher average BS-factor is more preferable.

Model-specific parameters are described in Models section.

5.2.3. Sample information

The following sample information is included in the result table:

- **ID**: internal sample number assigned by MoltenProt. Up to 96 samples can be processed at once (A1 to H12).
- **Capillary** (only NanoDSF data): capillary position in the device.
- **Condition**: annotation describing the contents of the sample. For NanoDSF data the value is read from sheet "Overview". Annotations can be changed via Layout editor.

6. Models

6.1. Overview

MoltenProt implements a variety of protein unfolding models, which should cover most common use-cases. All models rely on linear baseline extrapolation, which also means that data with sloping baselines should be processed with caution. This section contains a brief theoretical background for each model and introduces the recommended measure for final result ranking. All ranking measures are chosen in such a way that higher values correspond to higher stability of the protein.

6.2. Extensive and intensive readouts

Strictly speaking, the models implemented in MoltenProt are only applicable to extensive readouts, i.e. when the signal is proportional to protein concentration. While this is the case for raw fluorescence (330 or 350 nm) and Scattering, the F350/F330 Ratio is an intensive readout, because it is a proxy for the shape of the fluorescence spectrum. Applying equations for an extensive readout to an intensive readout produces an additional systematic error [2], [3]. The Ratio readout, however, tends to produce the most clean and easy to interpret sigmoidal curves, so omitting it from analysis decreases the explanatory power of the assay. For NanoDSF data MoltenProt also calculates deltaF readout (difference between fluorescence at 350 and 330 nm), which represents a trade-off between correctness and robustness. On the one hand, deltaF is an extensive readout, because it is a linear combination of F330 and F350. On the other hand, subtraction of fluorescences removes a significant part of the baseline drift and can make the unfolding transition more pronounced.

6.3. Equilibrium models

Equilibrium models rely on several assumptions: 1) protein unfolding is a reversible reaction; 2) at every timepoint of the measurement the system is at chemical equilibrium; 3) protein heat capacity change (ΔCp) is temperature-independent.

6.3.1. santoro1988

This model (applied to chemical denaturation) was initially described in ref. [4]. A more elaborate discussion for derivation of formulas is in ref. [3]. The model assumes that the protein exists in either native (N) or unfolded (U) state and there is an equilibrium between the folding and unfolding reactions (N ⇆ U). The law of signal F(T) is described by equation:

$$F(T)=\frac{k_N\cdot T+b_N+(k_U\cdot T+b_U)\cdot\exp(\frac{\Delta H_m}{R}\cdot(\frac{1}{T_m}-\frac{1}{T}))}{1+\exp(\frac{\Delta H_m}{R}\cdot(\frac{1}{T_m}-\frac{1}{T}))}$$

where kN, bN are slope and intercept of the pre-transition (native) baseline, kU bU are slope and intercept of post-transition baseline, R is the universal gas constant, ΔHm is enthalpy of unfolding at melting temperature Tm.

The final ranking metric is dG_std: Gibbs free energy of unfolding extrapolated to the standard state temperature (298.15 K). dG_std integrates the slope and the inflection point of an unfolding curve into a single measure.

By default extrapolation to the standard state temperature is linear, which is equal to the assumption that ΔCp is zero. If ΔCp is known, the extrapolated dG_std can be corrected by adding ΔCp * dCp_component. dCp_component is automatically calculated in santoro1988 mode and added to the output.

6.3.2. santoro1988i

This model is based on the same assumptions as the previous one, however, three states are possible for the protein: native (N), unfolded (U) and unfolding intermediate (I). If the Tm for N and I states is significantly different, it is possible to see two unfolding transitions in the experimental curve (two peaks on the smoothened derivative curve). Derivation is also described in ref [5]. The law of signal is as follows:

$$F(T)=\frac{k_N\cdot T+b_N+k_I\cdot\exp(\frac{\Delta H_{m1}}{R}\cdot(\frac{1}{T_1}-\frac{1}{T}))+(k_U\cdot T+b_U)\cdot\exp(\frac{\Delta H_{m1}}{R}\cdot(\frac{1}{T_1}-\frac{1}{T}))\cdot\exp(\frac{\Delta H_{m2}}{R}\cdot(\frac{1}{T_2}-\frac{1}{T}))}{1+\exp(\frac{\Delta H_{m1}}{R}\cdot(\frac{1}{T_1}-\frac{1}{T}))\cdot\exp(\frac{\Delta H_{m2}}{R}\cdot(\frac{1}{T_2}-\frac{1}{T}))}$$

where kN, bN are slope and intercept of the pre-transition (native) baseline, kU bU are slope and intercept of post-transition baseline, kI is the signal slope for the I state (the state is assumed to be short-lived, so the intercept is not modelled), R is the universal gas constant, ΔHm1 and ΔHm2 are enthalpy of unfolding at melting temperature T1 and T2 (melting temperature for N ⇆ I and I ⇆ U reactions).

The final ranking metric is dG_comb_std: Gibbs free energy of unfolding extrapolated to the standard state temperature (298.15 K), which is a sum of dG_std for reactions N ⇆ I and I ⇆ U (thermodynamic coupling).

6.4. Empirical models

Empirical models describe sigmoidal curves that are common in thermal unfolding assays without providing insights about the properties of the protein molecules.

6.4.1. santoro1988d

This model is a "descriptive" version of santoro1988 model: instead of enthalpy of unfolding at Tm (ΔHm), the model uses onset temperature T_onset to describe the steepness of the curve. T_onset is the temperature at which 1% of protein is unfolded. The exponent in santoro1988 model is thus substituted to the following expression:

$$\exp\left(\frac{(T-T_m)\cdot\log\left(\frac{0.01}{0.99}\right)}{T_{onset}-T_m}\right)$$

The final ranking metric is the square root of the sum of squared Tm and T_onset. This can be thought of as the Euclidean distance from the point 0,0 K of a scatter plot between Tm and T_onset. The samples that are most far away from this point will have the most beneficial combination of Tm and T_onset. This ranking assumes that Tm and T_onset are equally important for protein stability.

See also ref. [6].

6.4.2. santoro1988di

This model is a "descriptive" version of santoro1988i model. The exponents with ΔHm1-2 and Tm1-2 are substituted to exponents using T_onset1-2 and Tm1-2 (see santoro1988d). Similarly to santoro1988i, this model can describe unfolding curves with two transitions.

The final ranking metric is the sum of geometric means of Tm1 and T_onset1 and Tm2 and T_onset2.

6.5. Kinetic models

Kinetic models describe protein unfolding reactions via reaction rate constant, which links conversion of reactants to products with time. Arrhenius equation is used to model the temperature dependence of the reaction rate constant.

6.5.1. irrev

This model assumes that protein exists in states N and U only and the unfolding reaction is irreversible (N → U). The law of signal F(T) is defined as follows:

$$F(T)=k_U \cdot T+b_U+(k_N \cdot T+b_N) \cdot x_N(T)$$

where kN, bN are slope and intercept of the pre-transition (native) baseline, kU bU are slope and intercept of post-transition baseline and xN(T) is fraction of natively folded molecules as a function of temperature. xN(T) is obtained via numeric integration:

$$x_N(T)=\int_{T_{min}}^{T_{max}} \frac{-1}{v} \cdot \exp\left(\frac{-E_a}{R} \cdot \left(\frac{1}{T}-\frac{1}{T_f}\right)\right) \cdot x_N$$

where Tmin and Tmax are the start and end temperatures of the measurement, v is the scan rate (degrees/min), Ea is activation energy of unfolding, Tf is the temperature where reaction rate constant of unfolding (k) equals 1, R is the universal gas constant. xN is assumed to be 1 at Tmin. See refs [5], [7] for derivation of similar equations for differential scanning calorimetry data.

The final ranking metric for this model is the negative logarithm of the reaction rate constant at standard state temperature (pk_std). Similarly to dG_std in santoro1988, this metric integrates slope and inflection point of the unfolding curve (represented here as Ea and Tf) in a single measure of stability.

6.5.2. lumry_eyring

The Lumry-Eyring model [8] assumes that the protein exists in three states: native (N), unfolded (U) and aggregated (A). The reaction U → A is irreversible. Two more non-equilibrium reactions N → U and U → N with reaction rate constants kF and kR describe the transition from state N to state U. Fitting the whole model to typical experimental data is not stable, so in MoltenProt this model is applied in two steps:

1. Scattering data (part of NanoDSF datasets if the respective detector is available) is fit with irrev model to obtain activation energy Ea and Tf (temperature where the rate constant is 1) for reaction U → A. It is assumed that states and N and U produce the same Scattering signal.
2. The obtained parameters are supplied to the equation that describes protein unfolding signal F(T) (e.g. F350/F330 ratio in NanoDSF data) as a function of fraction native state xN, fraction unfolded state xU and fraction aggregated state xA.

$$F(T)=(k_N \cdot T+b_N) \cdot x_N+k_I * x_U+(kU \cdot T+b_U) \cdot x_A$$

$$x_N+x_U+x_A=1$$

where kN, bN are slope and intercept of the pre-transition (native) baseline, kU bU are slope and intercept of the post-transition baseline (which is in fact

represented by the A state, but not U), kI is the slope for the U state of unfolding. The equations for xU and xA are obtained with numeric integration [5].

The final ranking metric for this model is the negative logarithm of the ratio of the reaction rate constants for reactions N → U and U → N calculated at standard state temperature (pk_ratio_std). Similarly to dG_std in santoro1988, this metric integrates slope and inflection point of the unfolding curve in a single measure of stability. The special feature of lumry_eyring model in MoltenProt is that it can integrate not only the information from individual readouts, but also combine the stability as measured by Scattering with one selected unfolding readout.

7. Credits & copyright

7.1. MoltenProt


```
Copyright 2018,2019,2020 Vadim Kotov, Thomas C. Marlovits
    MoltenProt is free software: you can redistribute it and/or modify
    it under the terms of the GNU General Public License as published by
    the Free Software Foundation, either version 3 of the License, or
    (at your option) any later version.

    MoltenProt is distributed in the hope that it will be useful,
    but WITHOUT ANY WARRANTY; without even the implied warranty of
    MERCHANTABILITY or FITNESS FOR A PARTICULAR PURPOSE.  See the
    GNU General Public License for more details.

    You should have received a copy of the GNU General Public License
    along with MoltenProt.  If not, see <https://www.gnu.org/licenses/>.
```


7.2. Dependencies

7.2.1. pandas

https://pandas.pydata.org/


```
Copyright (c) 2008-2011, AQR Capital Management, LLC, Lambda Foundry, Inc. and
PyData Development Team
All rights reserved.

Copyright (c) 2011-2020, Open source contributors.

Redistribution and use in source and binary forms, with or without
modification, are permitted provided that the following conditions are met:

* Redistributions of source code must retain the above copyright notice, this
  list of conditions and the following disclaimer.

* Redistributions in binary form must reproduce the above copyright notice,
  this list of conditions and the following disclaimer in the documentation
  and/or other materials provided with the distribution.

* Neither the name of the copyright holder nor the names of its
  contributors may be used to endorse or promote products derived from
  this software without specific prior written permission.
```

THIS SOFTWARE IS PROVIDED BY THE COPYRIGHT HOLDERS AND CONTRIBUTORS "AS IS"
AND ANY EXPRESS OR IMPLIED WARRANTIES, INCLUDING, BUT NOT LIMITED TO, THE
IMPLIED WARRANTIES OF MERCHANTABILITY AND FITNESS FOR A PARTICULAR PURPOSE ARE
DISCLAIMED. IN NO EVENT SHALL THE COPYRIGHT HOLDER OR CONTRIBUTORS BE LIABLE
FOR ANY DIRECT, INDIRECT, INCIDENTAL, SPECIAL, EXEMPLARY, OR CONSEQUENTIAL
DAMAGES (INCLUDING, BUT NOT LIMITED TO, PROCUREMENT OF SUBSTITUTE GOODS OR
SERVICES; LOSS OF USE, DATA, OR PROFITS; OR BUSINESS INTERRUPTION) HOWEVER
CAUSED AND ON ANY THEORY OF LIABILITY, WHETHER IN CONTRACT, STRICT LIABILITY,
OR TORT (INCLUDING NEGLIGENCE OR OTHERWISE) ARISING IN ANY WAY OUT OF THE USE
OF THIS SOFTWARE, EVEN IF ADVISED OF THE POSSIBILITY OF SUCH DAMAGE.

7.2.2. numpy

http://numpy.org/

Copyright © 2005-2020, NumPy Developers.
All rights reserved.
Redistribution and use in source and binary forms, with or without modification,
are permitted provided that the following conditions are met:

* Redistributions of source code must retain the above copyright notice, this
  list of conditions and the following disclaimer.

* Redistributions in binary form must reproduce the above copyright notice,
  this list of conditions and the following disclaimer in the documentation
  and/or other materials provided with the distribution.

* Neither the name of the copyright holder nor the names of its
  contributors may be used to endorse or promote products derived from
  this software without specific prior written permission.

THIS SOFTWARE IS PROVIDED BY THE COPYRIGHT HOLDERS AND CONTRIBUTORS "AS IS"
AND ANY EXPRESS OR IMPLIED WARRANTIES, INCLUDING, BUT NOT LIMITED TO, THE
IMPLIED WARRANTIES OF MERCHANTABILITY AND FITNESS FOR A PARTICULAR PURPOSE ARE
DISCLAIMED. IN NO EVENT SHALL THE COPYRIGHT HOLDER OR CONTRIBUTORS BE LIABLE
FOR ANY DIRECT, INDIRECT, INCIDENTAL, SPECIAL, EXEMPLARY, OR CONSEQUENTIAL
DAMAGES (INCLUDING, BUT NOT LIMITED TO, PROCUREMENT OF SUBSTITUTE GOODS OR
SERVICES; LOSS OF USE, DATA, OR PROFITS; OR BUSINESS INTERRUPTION) HOWEVER
CAUSED AND ON ANY THEORY OF LIABILITY, WHETHER IN CONTRACT, STRICT LIABILITY,
OR TORT (INCLUDING NEGLIGENCE OR OTHERWISE) ARISING IN ANY WAY OUT OF THE USE
OF THIS SOFTWARE, EVEN IF ADVISED OF THE POSSIBILITY OF SUCH DAMAGE.

7.2.3. scipy

https://scipy.org/

Copyright © 2001, 2002 Enthought, Inc.
All rights reserved.

Copyright © 2003-2019 SciPy Developers.
All rights reserved.
Redistribution and use in source and binary forms, with or without modification,
are permitted provided that the following conditions are met:

- Redistributions of source code must retain the above copyright notice, this list of conditions and the following disclaimer.
- Redistributions in binary form must reproduce the above copyright notice, this list of conditions and the following disclaimer in the documentation and/or other materials provided with the distribution.
- Neither the name of Enthought nor the names of the SciPy Developers may be used to endorse or promote products derived from this software without specific prior written permission.

THIS SOFTWARE IS PROVIDED BY THE COPYRIGHT HOLDERS AND CONTRIBUTORS "AS IS" AND
ANY EXPRESS OR IMPLIED WARRANTIES, INCLUDING, BUT NOT LIMITED TO, THE IMPLIED
WARRANTIES OF MERCHANTABILITY AND FITNESS FOR A PARTICULAR PURPOSE ARE

DISCLAIMED. IN NO EVENT SHALL THE REGENTS OR CONTRIBUTORS BE LIABLE FOR ANY DIRECT, INDIRECT, INCIDENTAL, SPECIAL, EXEMPLARY, OR CONSEQUENTIAL DAMAGES (INCLUDING, BUT NOT LIMITED TO, PROCUREMENT OF SUBSTITUTE GOODS OR SERVICES; LOSS OF USE, DATA, OR PROFITS; OR BUSINESS INTERRUPTION) HOWEVER CAUSED AND ON ANY THEORY OF LIABILITY, WHETHER IN CONTRACT, STRICT LIABILITY, OR TORT (INCLUDING NEGLIGENCE OR OTHERWISE) ARISING IN ANY WAY OUT OF THE USE OF THIS SOFTWARE, EVEN IF ADVISED OF THE POSSIBILITY OF SUCH DAMAGE.

7.2.4. matplotlib

https://matplotlib.org/

"Copyright (c) 2012-2013 Matplotlib Development Team; All Rights Reserved"

7.2.5. PyQt5

https://www.riverbankcomputing.com/software/pyqt/

PyQt5 is copyright (c) Riverbank Computing Limited.
THERE IS NO WARRANTY FOR THE PROGRAM, TO THE EXTENT PERMITTED BY APPLICABLE LAW. EXCEPT WHEN OTHERWISE STATED IN WRITING THE COPYRIGHT HOLDERS AND/OR OTHER PARTIES PROVIDE THE PROGRAM "AS IS" WITHOUT WARRANTY OF ANY KIND, EITHER EXPRESSED OR IMPLIED, INCLUDING, BUT NOT LIMITED TO, THE IMPLIED WARRANTIES OF MERCHANTABILITY AND FITNESS FOR A PARTICULAR PURPOSE. THE ENTIRE RISK AS TO THE QUALITY AND PERFORMANCE OF THE PROGRAM IS WITH YOU. SHOULD THE PROGRAM PROVE DEFECTIVE, YOU ASSUME THE COST OF ALL NECESSARY SERVICING, REPAIR OR CORRECTION.

7.2.6. openpyxl

https://openpyxl.readthedocs.io/en/stable/

Copyright (c) 2010 openpyxl

Permission is hereby granted, free of charge, to any person obtaining a copy of this software and associated documentation files (the "Software"), to deal in the Software without restriction, including without limitation the rights to use, copy, modify, merge, publish, distribute, sublicense, and/or sell copies of the Software, and to permit persons to whom the Software is furnished to do so, subject to the following conditions:

The above copyright notice and this permission notice shall be included in all copies or substantial portions of the Software.

THE SOFTWARE IS PROVIDED "AS IS", WITHOUT WARRANTY OF ANY KIND, EXPRESS OR IMPLIED, INCLUDING BUT NOT LIMITED TO THE WARRANTIES OF MERCHANTABILITY, FITNESS FOR A PARTICULAR PURPOSE AND NONINFRINGEMENT. IN NO EVENT SHALL THE AUTHORS OR COPYRIGHT HOLDERS BE LIABLE FOR ANY CLAIM, DAMAGES OR OTHER LIABILITY, WHETHER IN AN ACTION OF CONTRACT, TORT OR OTHERWISE, ARISING FROM, OUT OF OR IN CONNECTION WITH THE SOFTWARE OR THE USE OR OTHER DEALINGS IN THE SOFTWARE.

Odict implementation in openpyxl/writer/odict.py uses the following licence:

Copyright (c) 2001-2011 Python Software Foundation
              2011 Raymond Hettinger
License: PYTHON SOFTWARE FOUNDATION LICENSE VERSION 2
         See http://www.opensource.org/licenses/Python-2.0 for full terms
Note: backport changes by Raymond were originally distributed under MIT license, but since the original license for Python is more restrictive than MIT, code cannot be released under its terms and still adheres to the limitations of Python license.

7.2.7. xlrd

https://pypi.org/project/xlrd/

There are two licenses associated with xlrd. This one relates to the bulk of the work done on the library::

```
Portions copyright © 2005-2009, Stephen John Machin, Lingfo Pty Ltd
All rights reserved.

Redistribution and use in source and binary forms, with or without
modification, are permitted provided that the following conditions are met:

1. Redistributions of source code must retain the above copyright notice,
this list of conditions and the following disclaimer.

2. Redistributions in binary form must reproduce the above copyright notice,
this list of conditions and the following disclaimer in the documentation
and/or other materials provided with the distribution.

3. None of the names of Stephen John Machin, Lingfo Pty Ltd and any
contributors may be used to endorse or promote products derived from this
software without specific prior written permission.

THIS SOFTWARE IS PROVIDED BY THE COPYRIGHT HOLDERS AND CONTRIBUTORS "AS IS"
AND ANY EXPRESS OR IMPLIED WARRANTIES, INCLUDING, BUT NOT LIMITED TO,
THE IMPLIED WARRANTIES OF MERCHANTABILITY AND FITNESS FOR A PARTICULAR
PURPOSE ARE DISCLAIMED. IN NO EVENT SHALL THE COPYRIGHT OWNER OR
CONTRIBUTORS
BE LIABLE FOR ANY DIRECT, INDIRECT, INCIDENTAL, SPECIAL, EXEMPLARY, OR
CONSEQUENTIAL DAMAGES (INCLUDING, BUT NOT LIMITED TO, PROCUREMENT OF
SUBSTITUTE GOODS OR SERVICES; LOSS OF USE, DATA, OR PROFITS; OR BUSINESS
INTERRUPTION) HOWEVER CAUSED AND ON ANY THEORY OF LIABILITY, WHETHER IN
CONTRACT, STRICT LIABILITY, OR TORT (INCLUDING NEGLIGENCE OR OTHERWISE)
ARISING IN ANY WAY OUT OF THE USE OF THIS SOFTWARE, EVEN IF ADVISED OF
THE POSSIBILITY OF SUCH DAMAGE.
```

This one covers some earlier work::

```
/*-
 * Copyright (c) 2001 David Giffin.
 * All rights reserved.
 *
 * Based on the the Java version: Andrew Khan Copyright (c) 2000.
 *
 *
 * Redistribution and use in source and binary forms, with or without
 * modification, are permitted provided that the following conditions
 * are met:
 *
 * 1. Redistributions of source code must retain the above copyright
 *    notice, this list of conditions and the following disclaimer.
 *
 * 2. Redistributions in binary form must reproduce the above copyright
 *    notice, this list of conditions and the following disclaimer in
 *    the documentation and/or other materials provided with the
 *    distribution.
 *
 * 3. All advertising materials mentioning features or use of this
 *    software must display the following acknowledgment:
 *    "This product includes software developed by
 *     David Giffin <david@giffin.org>."
 *
 * 4. Redistributions of any form whatsoever must retain the following
```

```
 *    acknowledgment:
 *    "This product includes software developed by
 *     David Giffin &lt;david@giffin.org&gt;."
 *
 * THIS SOFTWARE IS PROVIDED BY DAVID GIFFIN ``AS IS'' AND ANY
 * EXPRESSED OR IMPLIED WARRANTIES, INCLUDING, BUT NOT LIMITED TO, THE
 * IMPLIED WARRANTIES OF MERCHANTABILITY AND FITNESS FOR A PARTICULAR
 * PURPOSE ARE DISCLAIMED.  IN NO EVENT SHALL DAVID GIFFIN OR
 * ITS CONTRIBUTORS BE LIABLE FOR ANY DIRECT, INDIRECT, INCIDENTAL,
 * SPECIAL, EXEMPLARY, OR CONSEQUENTIAL DAMAGES (INCLUDING, BUT
 * NOT LIMITED TO, PROCUREMENT OF SUBSTITUTE GOODS OR SERVICES;
 * LOSS OF USE, DATA, OR PROFITS; OR BUSINESS INTERRUPTION)
 * HOWEVER CAUSED AND ON ANY THEORY OF LIABILITY, WHETHER IN CONTRACT,
 * STRICT LIABILITY, OR TORT (INCLUDING NEGLIGENCE OR OTHERWISE)
 * ARISING IN ANY WAY OUT OF THE USE OF THIS SOFTWARE, EVEN IF ADVISED
 * OF THE POSSIBILITY OF SUCH DAMAGE.
 *
```

7.2.8. joblib

https://joblib.readthedocs.io/

```
Copyright (c) 2008-2016, The joblib developers.
All rights reserved.

Redistribution and use in source and binary forms, with or without
modification, are permitted provided that the following conditions are met:

* Redistributions of source code must retain the above copyright notice, this
  list of conditions and the following disclaimer.

* Redistributions in binary form must reproduce the above copyright notice,
  this list of conditions and the following disclaimer in the documentation
  and/or other materials provided with the distribution.

* Neither the name of the copyright holder nor the names of its
  contributors may be used to endorse or promote products derived from
  this software without specific prior written permission.

THIS SOFTWARE IS PROVIDED BY THE COPYRIGHT HOLDERS AND CONTRIBUTORS "AS IS"
AND ANY EXPRESS OR IMPLIED WARRANTIES, INCLUDING, BUT NOT LIMITED TO, THE
IMPLIED WARRANTIES OF MERCHANTABILITY AND FITNESS FOR A PARTICULAR PURPOSE ARE
DISCLAIMED. IN NO EVENT SHALL THE COPYRIGHT HOLDER OR CONTRIBUTORS BE LIABLE
FOR ANY DIRECT, INDIRECT, INCIDENTAL, SPECIAL, EXEMPLARY, OR CONSEQUENTIAL
DAMAGES (INCLUDING, BUT NOT LIMITED TO, PROCUREMENT OF SUBSTITUTE GOODS OR
SERVICES; LOSS OF USE, DATA, OR PROFITS; OR BUSINESS INTERRUPTION) HOWEVER
CAUSED AND ON ANY THEORY OF LIABILITY, WHETHER IN CONTRACT, STRICT LIABILITY,
OR TORT (INCLUDING NEGLIGENCE OR OTHERWISE) ARISING IN ANY WAY OUT OF THE USE
OF THIS SOFTWARE, EVEN IF ADVISED OF THE POSSIBILITY OF SUCH DAMAGE.
```

7.2.9. Oxygen icons

https://github.com/KDE/oxygen-icons

```
The Oxygen Icon Theme
    Copyright (C) 2007 Nuno Pinheiro &lt;nuno@oxygen-icons.org&gt;
    Copyright (C) 2007 David Vignoni &lt;david@icon-king.com&gt;
    Copyright (C) 2007 David Miller &lt;miller@oxygen-icons.org&gt;
    Copyright (C) 2007 Johann Ollivier Lapeyre &lt;johann@oxygen-icons.org&gt;
    Copyright (C) 2007 Kenneth Wimer &lt;kwwii@bootsplash.org&gt;
    Copyright (C) 2007 Riccardo Iaconelli &lt;riccardo@oxygen-icons.org&gt;


and others
```

This library is free software; you can redistribute it and/or
modify it under the terms of the GNU Lesser General Public
License as published by the Free Software Foundation; either
version 3 of the License, or (at your option) any later version.

This library is distributed in the hope that it will be useful,
but WITHOUT ANY WARRANTY; without even the implied warranty of
MERCHANTABILITY or FITNESS FOR A PARTICULAR PURPOSE. See the GNU
Lesser General Public License for more details.

You should have received a copy of the GNU Lesser General Public
License along with this library. If not, see <http://www.gnu.org/licenses/>.

Clarification:

The GNU Lesser General Public License or LGPL is written for
software libraries in the first place. We expressly want the LGPL to
be valid for this artwork library too.

KDE Oxygen theme icons is a special kind of software library, it is an
artwork library, it's elements can be used in a Graphical User Interface, or
GUI.

Source code, for this library means:
- where they exist, SVG;
- otherwise, if applicable, the multi-layered formats xcf or psd, or
otherwise png.

The LGPL in some sections obliges you to make the files carry
notices. With images this is in some cases impossible or hardly useful.

With this library a notice is placed at a prominent place in the directory
containing the elements. You may follow this practice.

The exception in section 5 of the GNU Lesser General Public License covers
the use of elements of this art library in a GUI.

kde-artists [at] kde.org

8. References

[1] A. D. Robertson and K. P. Murphy, “Protein Structure and the Energetics of Protein Stability,” *Chem. Rev.*, vol. 97, no. 5, pp. 1251–1268, Aug. 1997, doi: 10.1021/cr960383c.

[2] E. Monsellier and H. Bedouelle, “Quantitative measurement of protein stability from unfolding equilibria monitored with the fluorescence maximum wavelength,” *Protein Eng Des Sel*, vol. 18, no. 9, pp. 445–456, Sep. 2005, doi: 10.1093/protein/gzi046.

[3] H. Bedouelle, “Principles and equations for measuring and interpreting protein stability: From monomer to tetramer,” *Biochimie*, vol. 121, pp. 29–37, Feb. 2016, doi: 10.1016/j.biochi.2015.11.013.

[4] M. M. Santoro and D. W. Bolen, “Unfolding free energy changes determined by the linear extrapolation method. 1. Unfolding of phenylmethanesulfonyl .alpha.-chymotrypsin using different denaturants,” *Biochemistry*, vol. 27, no. 21, pp. 8063–8068, Oct. 1988, doi: 10.1021/bi00421a014.

[5] S. Mazurenko, A. Kunka, K. Beerens, C. M. Johnson, J. Damborsky, and Z. Prokop, “Exploration of Protein Unfolding by Modelling Calorimetry Data

from Reheating," *Sci Rep*, vol. 7, Nov. 2017, doi: 10.1038/s41598-017-16360-y.
[6] V. Kotov *et al.*, "High-throughput stability screening for detergent-solubilized membrane proteins," *Scientific Reports*, vol. 9, no. 1, p. 10379, Jul. 2019, doi: 10.1038/s41598-019-46686-8.
[7] J. M. Sanchez-Ruiz, "Theoretical analysis of Lumry-Eyring models in differential scanning calorimetry," *Biophys J*, vol. 61, no. 4, pp. 921–935, Apr. 1992.
[8] R. Lumry and H. Eyring, "Conformation Changes of Proteins," *J. Phys. Chem.*, vol. 58, no. 2, pp. 110–120, Feb. 1954, doi: 10.1021/j150512a005.
