# Supplementary material for: In‐depth interrogation of protein thermal unfolding data with MoltenProt
Source: Protein Sci. 2020 Nov 21;30(1):201–17. doi: 10.1002/pro.3986 (PMC7737771; doi:10.1002/pro.3986)
Supplement: Supplementary file 2 — Supplementary code. [file PRO-30-201-s002.zip › moltenprot/resources/report.template]

Thermostability Report for $FILE


$HEATMAP\_TABLE

Sample:


$BUTTONS

  

This report was generated with MoltenProt v. $VERSION on $TIMESTAMP.
*If you found this software helpful, please cite:*
Kotov et al., Biophysical Journal. 116, 191a (2019)
