# Supplementary figures and images for: In‐depth interrogation of protein thermal unfolding data with MoltenProt
Source: Protein Sci. 2020 Nov 21;30(1):201–17. doi: 10.1002/pro.3986 (PMC7737771; doi:10.1002/pro.3986)

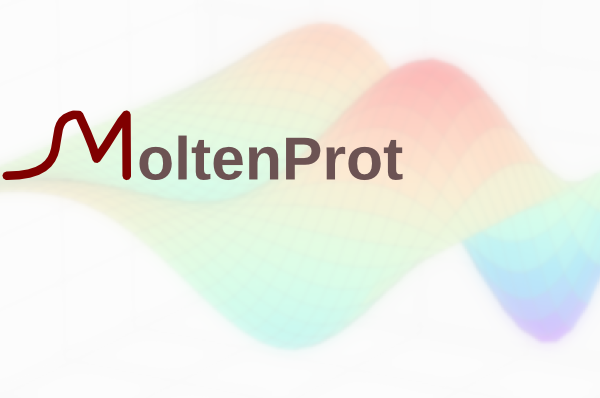

Supplement: Supplementary file 2 — Supplementary code. [file PRO-30-201-s002.zip › moltenprot/ui/splashscreen.png]

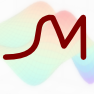

Supplement: Supplementary file 2 — Supplementary code. [file PRO-30-201-s002.zip › moltenprot/ui/icons/app_icon.png]

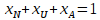

Supplement: Supplementary file 2 — Supplementary code. [file PRO-30-201-s002.zip › moltenprot/doc/index_html_53b9db394b61367c.gif]

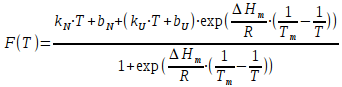

Supplement: Supplementary file 2 — Supplementary code. [file PRO-30-201-s002.zip › moltenprot/doc/index_html_8a1ff70f467fcc1c.gif]

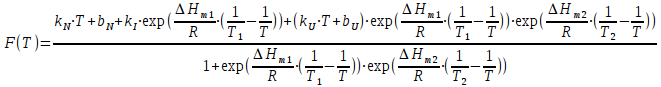

Supplement: Supplementary file 2 — Supplementary code. [file PRO-30-201-s002.zip › moltenprot/doc/index_html_165f619840c03589.gif]

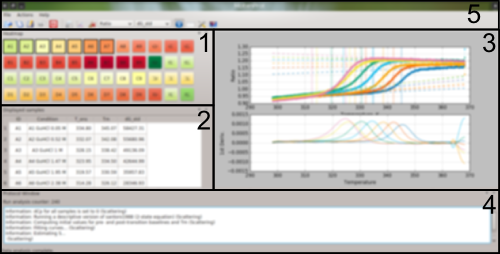

Supplement: Supplementary file 2 — Supplementary code. [file PRO-30-201-s002.zip › moltenprot/doc/MainWindow.png]

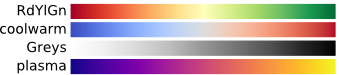

Supplement: Supplementary file 2 — Supplementary code. [file PRO-30-201-s002.zip › moltenprot/doc/colormaps.png]

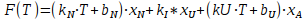

Supplement: Supplementary file 2 — Supplementary code. [file PRO-30-201-s002.zip › moltenprot/doc/index_html_1a64340c1caaafdc.gif]

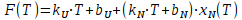

Supplement: Supplementary file 2 — Supplementary code. [file PRO-30-201-s002.zip › moltenprot/doc/index_html_b7d5b4664cef503e.gif]

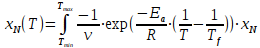

Supplement: Supplementary file 2 — Supplementary code. [file PRO-30-201-s002.zip › moltenprot/doc/index_html_693cf9b4f65b9717.gif]

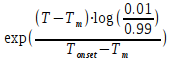

Supplement: Supplementary file 2 — Supplementary code. [file PRO-30-201-s002.zip › moltenprot/doc/index_html_b873ef725266e963.gif]
